# Ruthenium-centred btp glycoclusters as inhibitors for *Pseudomonas aeruginosa* biofilm formation†

**DOI:** 10.1039/d0ra05107a

**Published:** 2021-05-04

**Authors:** Ciaran O'Reilly, Salvador Blasco, Bina Parekh, Helen Collins, Gordon Cooke, Thorfinnur Gunnlaugsson, Joseph P. Byrne

**Affiliations:** School of Chemistry, Trinity Biomedical Sciences Institute, Trinity College Dublin Ireland; School of Medicine, University College Dublin Belfield Dublin 4 Ireland; Department of Applied Science, Tallaght Campus, Technological University Dublin Ireland; School of Chemistry, National University of Ireland Galway University Road Galway Ireland joseph.byrne@nuigalway.ie

## Abstract

Carbohydrate-decorated clusters (glycoclusters) centred on a Ru(ii) ion were synthesised and tested for their activity against *Pseudomonas aeruginosa* biofilm formation. These clusters were designed by conjugating a range of carbohydrate motifs (galactose, glucose, mannose and lactose, as well as galactose with a triethylene glycol spacer) to a btp (2,6-bis(1,2,3-triazol-4-yl)pyridine) scaffold. This scaffold, which possesses a *C*_2_ symmetry, is an excellent ligand for d-metal ions, and thus the formation of the Ru(ii)-centred glycoclusters 7 and 8Gal was achieved from 5 and 6Gal; each possessing four deprotected carbohydrates. Glycocluster 8Gal, which has a flexible spacer between the btp and galactose moieties, showed significant inhibition of *P. aeruginosa* bacterial biofilm formation. By contrast, glycocluster 7, which lacked the flexible linker, didn't show significant antimicrobial effects and neither does the ligand 6Gal alone. These results are proposed to arise from carbohydrate–lectin interactions with LecA, which are possible for the flexible metal-centred multivalent glycocluster. Metal-centred glycoclusters present a structurally versatile class of antimicrobial agent for *P. aeruginosa*, of which this is, to the best of our knowledge, the first example.

## Abstract 2

Ruthenium-centred glycoclusters based on carbohydrate-functionalised bis(triazolyl)pyridine ligands show *Pseudomonas aeruginosa* biofilm inhibition, with activity that is dependent on ligand structure.

## Graphical abstract

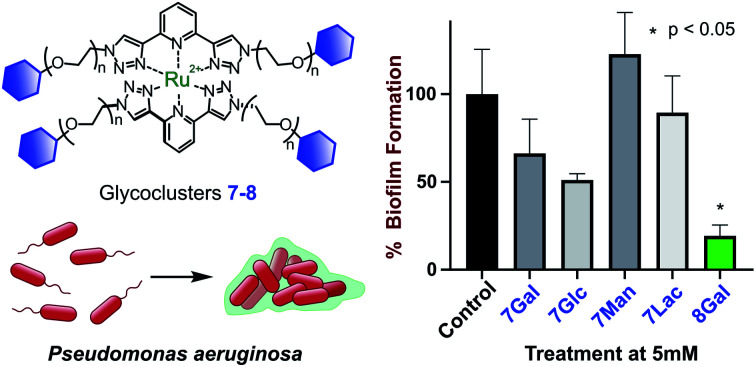

## Introduction

*Pseudomonas aeruginosa* is a ubiquitous Gram-negative pathogenic bacteria, which is of great medical significance as a result of its multidrug resistance and effects on immune-compromised patients, such as those suffering from cystic fibrosis. It has been identified as one of the ‘ESKAPE’ pathogens, which are the cause of many healthcare-acquired infections, and for which development of new antimicrobial agents is vital.^1^ Biofilm formation is a key part of pathogenic behaviour for many bacteria, and its inhibition is an important target.^2–4^*P. aeruginosa* expresses two carbohydrate-binding proteins on its surface which have been identified as essential to its pathogenesis, namely lectins LecA and LecB (also known as PA-IL and PA-IIL).^5,6^ These lectins are pivotal in processes including adhesion to epithelial and endothelial cells, as well as biofilm formation. Biofilms are structured aggregates of bacteria linked together by these lectins and encapsulated in a complex extracellular matrix,^2,7^ and their formation is implicated in chronic *P. aeruginosa* infections, which exhibit marked resistance to antibiotics and the capacity to evade host defences.^8^ The physical barrier of the biofilm may be sufficient to prevent or slow penetration by conventional antimicrobial agents, which are effective on the bacteria in its planktonic form. Novel compounds that prevent biofilm-based infection without directly killing the bacteria could restore sensitivity to established antibiotics.

These surface lectins, LecA and LecB, show specific binding for galactosides and fucosides respectively and thus targeting these proteins with carbohydrate-appended therapeutic molecules is a valuable strategy (*e.g.* for disrupting biofilm formation).^9–12^ In 2008, Hauber *et al.* showed that inhalation of galactose and fucose monosaccharides was a safe treatment, and subsequently research has focussed on developing more selective or potent antimicrobial agents.^9,13–15^ Carbohydrate multivalency has been shown to be very important for providing high-affinity interactions, represented by a range glycopeptide dendrimers, glyco-nanoparticles and glycoclusters, including compounds built upon macrocyclic scaffolds.^11,15–22^

A recent example of such an antiadhesive glycocluster system, described by Vidal and co-workers, was based on a calix[4]arene scaffold, that was derivatised with galactose and fucose monosaccharide units.^19^ These carbohydrates selectively bind LecA and LecB and, as a result, this macrocyclic system was shown to inhibit biofilm formation, and was also studied in an *in vivo* mouse model where it was shown to demonstrate protection against *P. aeruginosa* lung infection. The topology of carbohydrate presentation from the calix[4]arene scaffold was shown to play an important role, with tetravalent clusters presenting two pairs of epitopes from opposite faces of the calix[4]arene scaffold proving most potent.^23,24^ In this example, triethylene glycol was used successfully as a flexible spacer, however different spacers, both rigid and flexible, have also been utilised to allow for tuning of avidity of lectin inhibitors.^10,14,25,26^ In order to present the saccharide units in a three-dimensional way, various different scaffold-types have been utilised to design ligands for LecA, including glycopeptide dendrimer, cyclodextrins and carbohydrate-based scaffolds. To the best of our knowledge, coordination of a metal ion has yet to be exploited in design of inhibitors targeting the *P. aeruginosa* lectins. Such a strategy would facilitate access to three-dimensional geometries of carbohydrate epitopes defined by the metal coordination geometry, and allow for immediate increase of multivalency of a ligand system, upon coordinating multiple ligands to the same metal centre, providing complexes with potential to inhibit biofilm formation by the bacteria. Cationic charge has also been shown to be a characteristic of many antimicrobial dendrimers, which can disrupt bacterial membranes; this presents another possible added advantage to introducing metal ions into glycocluster structures for targeting bacteria.^27–29^

Ligands containing the glycosyl-triazolyl-pyridyl motif have come to light in recent years, mostly applied as ligands for catalysis,^30–32^ and enzyme inhibitors.^33,34^ The only metal complexes reported with such promising ligands are Pd(ii)-containing catalysts for C–C coupling reactions^35,36^ and Ru(ii) complexes.^37^ We have an interest in triazole ligands, such as those based on the 2,6-bis(1,2,3-triazol-4-yl)pyridine (btp) motif.^38^ Having exploited such ligands to coordinate transition metal ions including Ru(ii), Ni(ii), Ir(iii) and Pt(ii)^39^ as well as forming luminescent self-assemblies and MOFs with lanthanide ions,^40–44^ and mechanically interlocked molecules,^45^ we set out to investigate the use of btp systems as potential antimicrobial agents. In previous studies, we and others have crystallographically demonstrated that Ru(ii) forms 1 : 2 complexes with btp ligands, which would allow for the incorporation of a minimum of four glycans into a single complex.^38,39,46–48^ In this article, we describe a series of Ru(ii)-centred tetravalent glycoclusters, synthesised from carbohydrate-derived btp ligands, and assess their suitability as inhibitors for *P. aeruginosa* biofilm formation.

## Results and discussion

### Synthesis of ligands and Ru(ii) complexes

**Scheme 1:**
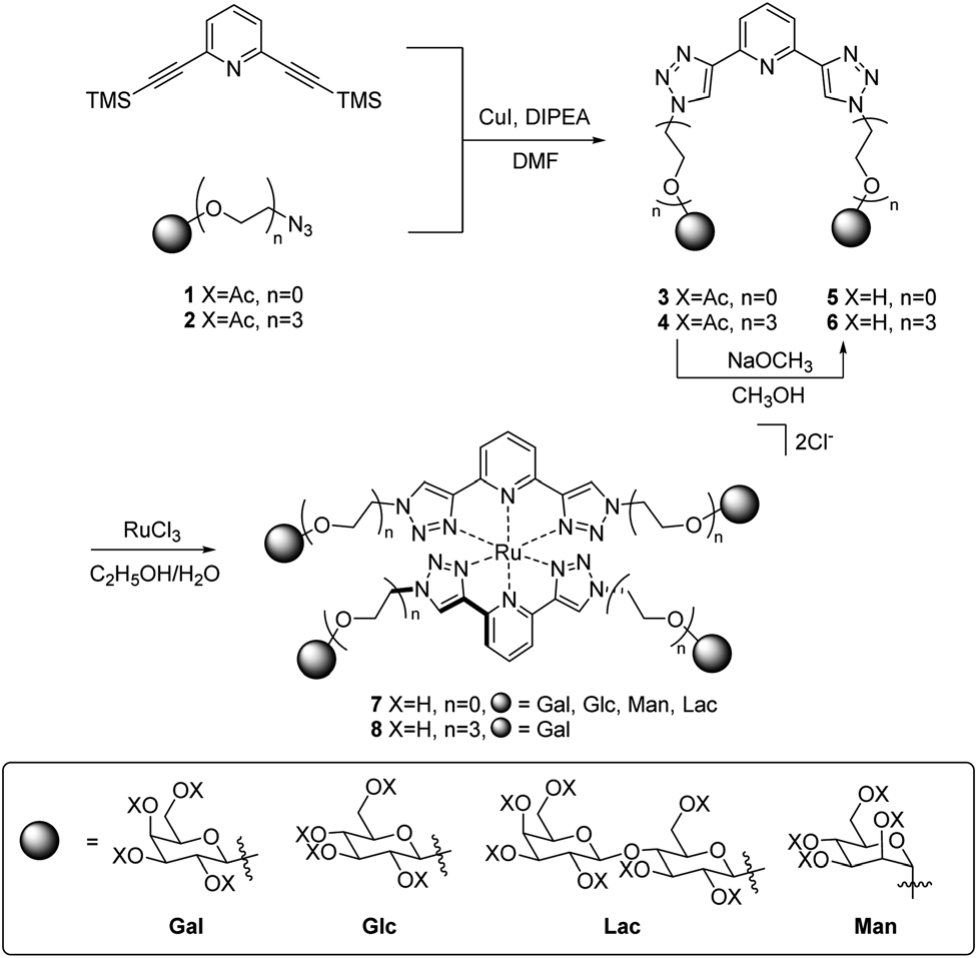
Synthesis of Ru(ii)-centred glycoclusters 7 and 8Gal.

The synthesis of acetyl-protected btp compounds, hydrolysed ligands, and target Ru(ii) complexes are shown in Scheme 1. The CuAAC reaction is typically used to prepare btp ligands, and as such azide functionality was introduced to monosaccharides. Stereoselective azidation of peracetylated glycosides *via* treatment with TMS-N_3_ under Lewis acid catalysed conditions gave access to azide precursors 1,^49^ while 2Gal was synthesised from a chloro-precursor, as described in the literature.^50^ Triethylene glycol spaced galactosides have previously been included in calix[4]arene-based inhibitors for LecA, and as such were also included in the family of new compounds designed here.^19,23^ These carbohydrate azides were then used as substrates for the CuAAC reaction with 2,6-bis(TMS-ethynyl)pyridine, yielding protected btp compounds 3 in good yields (63–82%) and 4Gal in 79% yield. The ^1^H NMR spectra of triazole compounds each displayed a single set of resonances, corresponding to a single anomer, for instance with doublets observed at 6.40 ppm (*J* = 9.2 Hz) for β-anomer 3Gal,^32^ and 4.48 ppm (*J* = 8.0 Hz) for β-anomer 4Gal. HRMS analysis further supported the formation of the proposed structures (see Experimental and ESI† for full characterisation).

**Fig. 1:**
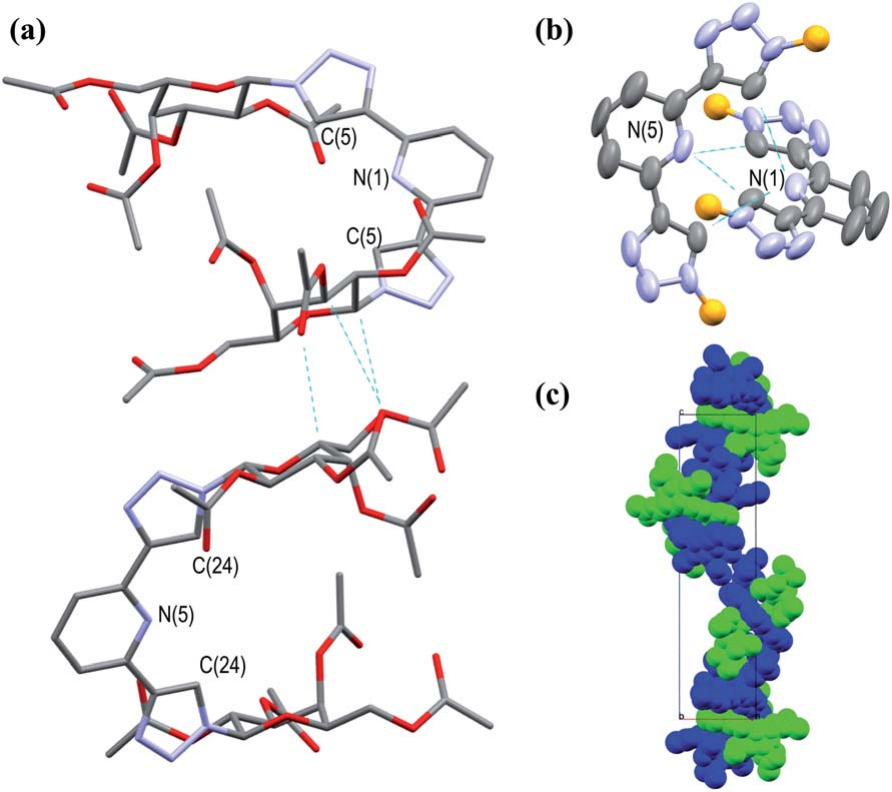
Projections of the X-ray crystal structure of 3Gal,‡‡Selected crystallographic and refinement data for crystal of 3Gal: *a*, *b*, *c* (Å): 13.033(15), 13.033(15), 44.95(5); *α*, *β*, *γ* (°): 90, 90, 120; *V* (Å^3^): 6613(17); *Z*: 6; *F*(000): 2748; *D*_c_ (Mg m^−3^): 1.316; *μ* (mm^−1^): 0.91; GOF, *R*_1_, w*R*_2_, flack: 1.075, 0.062, 0.151, 0.03(9). showing various features of the structure (hydrogen atoms omitted for clarity, CCDC deposition number 2004944†): (a) capped stick model showing the two distinct molecules in the structure packing due to weak supramolecular interactions between the adjacent sugar moieties; (b) thermal ellipsoid model showing the non-classical hydrogen bonding interactions between the two btp motifs; the carbohydrate arms are omitted for clarity (represented as orange spheres); (c) a space-filling model, showing the packing of the molecules with a *C*3_2_ screw-axis.

In addition to the above characterisation of the acetyl protected ligands, single crystals of 3Gal were grown by slow diffusion of (CH_3_)_2_CHOH into a DMSO-*d*_6_ solution as colourless hexagonal plates, which were found to be suitable for X-ray diffraction analysis. The ligand crystallised in the trigonal space group *P*3_2_21. The asymmetric unit contains two distinct residues, each being half a molecule of 3Gal, the other half of each molecule being generated by symmetry. These two complete molecules are shown in Fig. 1(a). The btp motifs of the two crystallographically distinct molecules were also shown to intertwine by non-classical hydrogen bonding interactions between the triazolyl C–H and the pyridyl nitrogen atoms of the other btp motif, with hydrogen bond lengths of C–H⋯N = 3.511(10) and 3.482(9) Å, Fig. 1(b). This dimeric interaction matched our previous observations, which have been exploited to form interlocked supramolecular architectures.^40,45^

Crystallographic packing analysis also showed weak supramolecular interactions between the carbohydrate moieties of the adjacent molecules, causing them to stack in the manner seen in Fig. 1(c); all C–H⋯O distances were of the order of 3.5 Å and are shown in a table in ESI.† The triazole rings of btp were found to be slightly twisted out of the plane of the pyridyl rings by *ca.* 13° in this elegant structure. The unit cell of this crystal structure contained 6 molecules (*Z* = 6); the two molecules shown in Fig. 1(a) repeat in a left-handed 3_2_ screw axis, Fig. 1(c).

**Fig. 2:**
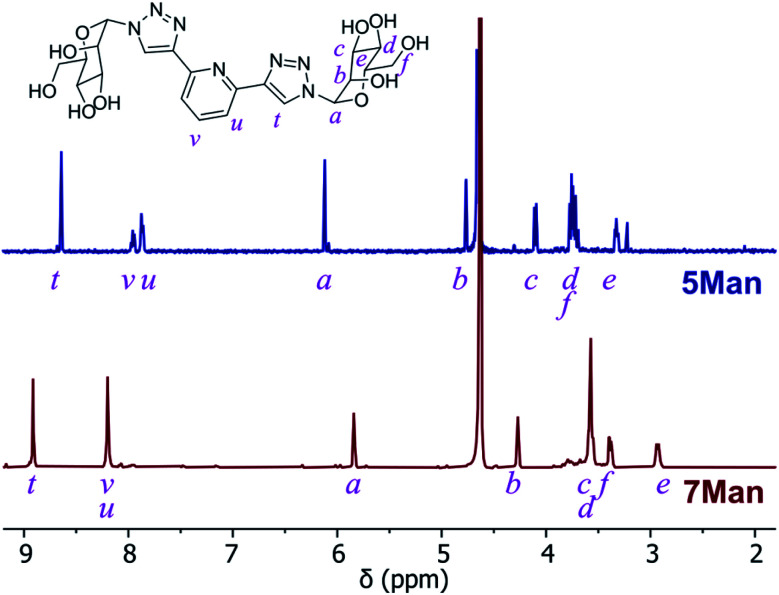
^1^H NMR spectra (D_2_O) of btp ligand 5Man (top) and the resulting Ru(ii) complex 7Man (bottom).

*O*-Deacetylation under Zemplén conditions gave access to the fully deprotected carbohydrate derivatives 5 and 6Gal in good yields (63–78%), which were soluble in aqueous media. ^1^H NMR spectra confirmed the removal of acetyl groups (note the absence of proton resonances at *ca.* 2 ppm in Fig. 2) and HRMS analysis was also consistent with the formation of btp structures decorated with unprotected saccharide moieties.

The corresponding Ru(ii) complexes 7 and 8Gal were prepared upon heating 2 equivalents of the ligand (5 or 6Gal) with RuCl_3_·3H_2_O in aqueous ethanol solution under microwave irradiation at 120 °C for 40 minutes. Complexes 7 and 8Gal, of the general structure [Ru·(L)_2_]Cl_2_, were formed as a single species. Changes observed in the ^1^H NMR spectrum (400 MHz, D_2_O) of 7Man, for instance (Fig. 2), including a shift in the resonance arising from the triazolyl CH from 8.35 to 8.91 ppm, and the coalescence of the proton resonances from the pyridyl ring into a multiplet centred at 8.2 ppm, are indicative of coordination of Ru(ii) by the btp ligand. HRMS analysis gave signals corresponding to the doubly charged [M−2Cl]^2+^ ion in all cases. Single crystals suitable for X-ray diffraction were not obtained for these complexes, but based on known structures of related [Ru(btp)_2_]X_2_ complexes, it is anticipated that the two tridentate btp ligands coordinate Ru(ii) in an approximately octahedral geometry,^38,39,47,51^ and as such the carbohydrate epitopes from each divalent ligand would be presented orthogonally.

### Biofilm inhibition

To the best of our knowledge, only a few ruthenium complexes have reported significant inhibition of biofilm formation by *P. aeruginosa*,^52–55^ with these usually being bimetallic complexes. However in general Ru(ii)–polypyridyl complexes have shown lower activity towards Gram-negative species (particularly *P. aeruginosa*) when compared to Gram-positive bacteria (such as MRSA).^55–57^

**Fig. 3:**
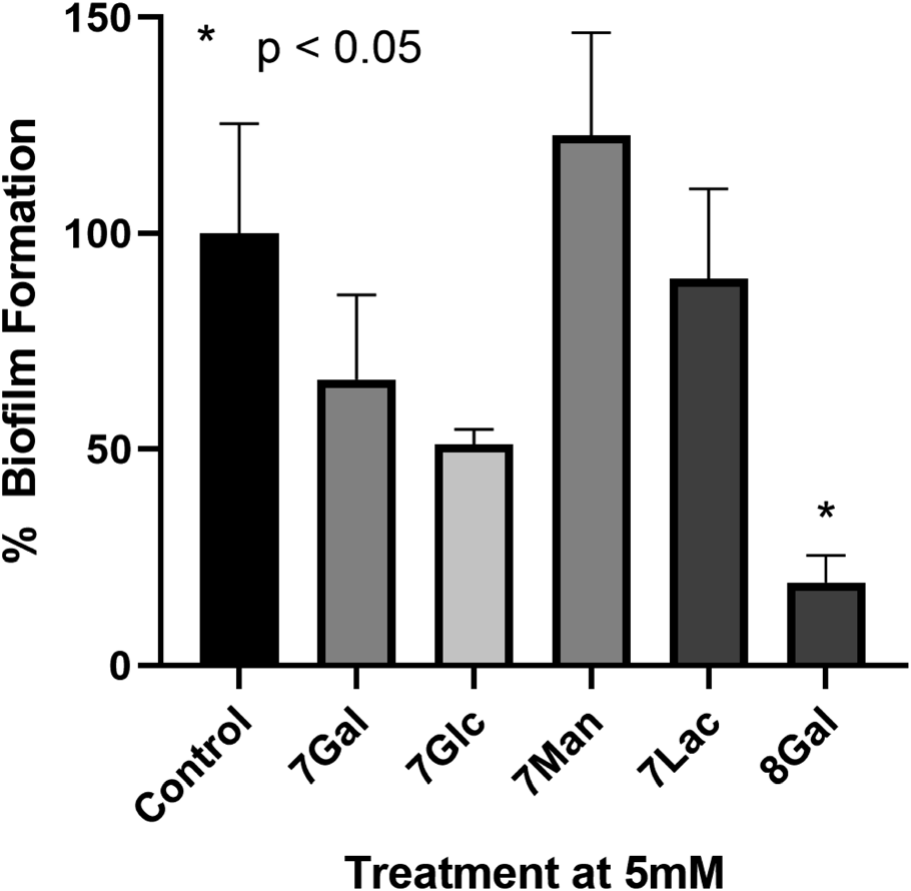
Percentage biofilm formation determined by crystal violet staining of biofilms upon incubation of *P. aeruginosa* with 5 mM of a range of Ru(ii) glycoclusters. Decreased absorbance at 590 nm indicated inhibition of biofilm formation. One way ANOVA on ranks performed, *p < 0.05 *versus* control.

In order to determine the potential of Ru(ii) glycoclusters 7 and 8Gal to inhibit bacterial biofilm formation, samples of *P. aeruginosa* (PAO1) were incubated with 5 mM of Ru(ii) complexes for 24 hours and the biofilm biomass determined by staining with crystal violet. Inhibition was characterised by decrease in absorbance at 590 nm, compared to control experiments in the absence of any glycocluster (see Fig. 3). None of the tested complexes were either bacteriostatic nor bactericidal to PAO1 at these concentrations (see ESI† for MIC and MBC data).

When complexes 7, where the carbohydrates are directly bonded to the triazole moieties, were incubated with *P. aeruginosa*, none of the Ru(ii) complexes had statistically significant impact on biofilm formation, regardless of the identity of the carbohydrate. This would suggest that the saccharide motifs were not able to interact with the target lectin in this configuration, either due to lack of flexibility, or due to the distances between carbohydrate units not matching the distance between lectin carbohydrate-binding sites. When designing LecA inhibitors, Pieters and co-workers describe a separation of 26 Å between the carbohydrates in neighbouring binding sites of the lectin,^14,25,58^ and while X-ray crystal structures of Ru(ii) complexes 7 were not obtained, the analogous galactose–galactose distance in ligand 3Gal, measured between the anomeric carbons, was significantly shorter (*ca.* 8 Å). Based on analogy to previously-reported octahedral Ru(ii) btp complexes, the distance between anomeric carbons in complexes 7 would be expected to be 8–11 Å. Hence, it is possible that this significant deviation from the ideal multivalent binding geometry for LecA explains the lack of interaction, even with a carbohydrate epitope known to interact with the lectin.

Complex 8Gal, on the other hand, where triethylene glycol chains confer the structure with further flexibility,^19,25,59^ demonstrated significant inhibition of biofilm formation, with *ca.* 80% decrease *versus* the control (*p* < 0.05). Although highly-multivalent glyconanoparticles have been able to achieve these results at much lower concentrations (μM),^22^ the activity of 8Gal compares favourably with other similar-valency calix[4]arene and triazine-based glycoclusters, which also showed statistically significant biofilm reduction at 5 mM concentration.^13,19^ The ligand 6Gal alone does not lead to biofilm inhibition under analogous conditions (see ESI, Fig. S28†), suggesting that the multivalent glycocluster structure is required for this effect to be observed.

The striking difference in behaviour between complexes 7 and 8Gal, all of which are cationic Ru(ii)-containing compounds, suggests that antimicrobial properties of this complex do not arise simply from the presence of the cationic metal ion. Nor is the ligand structure alone responsible for the effect on *P. aeruginosa* biofilm formation. Rather, the combination of the flexible ligand and the mode of presentation of the carbohydrate units in the tetravalent cluster structure is proposed to be the origin of the observed effect. This result confirms the potential of using metal ions as scaffolds for the construction of antimicrobial multivalent glycoclusters from ligands of lower valency, giving rise to biofilm inhibition activity which is not primarily caused by the metal ion. This will form a basis for future developments, which could build on the variety of geometric structures available through coordination chemistry of alternative d- and f-metal ions to explore the relationship between complex topology and biofilm inhibition in detail.

## Conclusions

We have successfully synthesised a range of protected carbohydrate-derived tridentate btp ligands and characterised them, including by single crystal X-ray diffraction in the case of 3Gal. These ligands were conveniently deprotected under Zemplén conditions and formed Ru(ii) complexes of the form [Ru·(L)_2_]Cl_2_, which were tetravalent glycoclusters.

Complex 8Gal, containing flexible triethylene glycol spacers between the btp and galactose moieties, showed significant inhibition of *P. aeruginosa* biofilm formation at 5 mM concentration, while neither complexes 7 nor ligand 6Gal had an impact on biofilm formation under the same conditions. None of the complexes were bacteriostatic or bactericidal. This behaviour is rationalised in terms of the known affinity of soluble lectin LecA for galactose and the advantages of multivalency reported in the literature for inhibitors of this protein that are able to span adjacent carbohydrate-binding sites. As such, it is clear that the topology of presentation of carbohydrate units, templated by coordination chemistry plays an important role in antimicrobial activity of such metal-centred glycoclusters.

These results indicate that coordination chemistry may be used to conveniently direct the topology of multivalent glycoclusters, built up from simpler lower-valency building block ligands. As the Ru(ii) centre does not appear to play a significant role in any antimicrobial properties of 7 or 8Gal, it is conceivable that other metal ions could easily be used, either for increased economy, or to exploit complementary properties like magnetism or luminescence (*e.g.* for use in applications such as imaging of biofilms). We will further develop on this work by investigating the biofilm inhibition properties, and impact on biofilm-related antibiotic resistance, of related systems with differing geometries, metal centres, and spacers in future studies.

## Experimental

### Materials and methods

All chemicals and reagents were purchased from commercial sources and used without further purification. Electrospray mass spectra (ESI) were acquired using a Micromass time of flight mass spectrometer (TOF), interfaced to a Waters 2690 HPLC or a Waters LCT Premiere XE (with leucine enkephalin used as an internal lock mass). MALDI Q-Tof mass spectra were carried out on a MALDI Q-Tof Premier (Waters Corporation, Micromass MS technologies, Manchester, UK) and high-resolution mass spectrometry was performed using Glu-Fib as an internal reference. NMR spectra were recorded on a 400 MHz Bruker Avance III spectrometer or a 500 MHz Agilent spectrometer. Chemical shifts expressed in parts per million (ppm/*δ*) are reported relative to internal tetramethylsilane in CDCl_3_ or CD_3_OD or relative to HOD in D_2_O. Coupling constants (*J*) are expressed in Hz. Infrared spectra were recored on a PerkinElmer Spectrum 100 or 400 FT-IR spectrometer with universal ATR sampling accessory. All microwave reactions were carried out Biotage Microwave Vials in a Biotage Initiator Eight EXP microwave reactor.

X-ray diffraction data for 3Gal was measured on a Bruker Apex2 Duo using a high intensity Cu-Kα radiation source (*λ* = 1.54178 Å). The datasets were collected and processed using Bruker APEX3 suite of programs. All structures were solved by direct methods (SHELXS-2018/3) and refined against all *F*^2^ data (SHELXL-2018/3).^60^ All H-atoms, were positioned geometrically and refined using a riding model.

### Biofilm formation assay

Biofilm formation assays were used based on the methodology from previous published work.^61^ In brief, starting from an overnight liquid culture, a dilution containing approximately 10^8^ CFUs mL^−1^ was made of *Pseudomonas aeruginosa*, PAO1 strain (a kind donation from Prof. Seamas Donnelly, School of Medicine, Trinity College Dublin). For each biofilm experiment, 8 wells of a round-bottomed polypropylene 96-well micro plate (Corning® Costar® purchased from Sigma Aldrich, Dublin Cat#CLS3596) were inoculated with 100 μl of this dilution, 8 wells were inoculated with the dilution and treated with 5 mM of the Ru(ii) glycoclusters and 8 control wells were filled with sterile medium (bacteria alone). Following 4 hours of adhesion, the supernatant, containing non-adhered cells, was removed from each well and plates rinsed using phosphate buffered saline (PBS) solution. Following this 100 μl of fresh media was added to the control wells and fresh media with 5 mM of the Ru(ii) glycoclusters was added to the appropriate wells, the plate was then incubated for a further 24 hours. After 24 hours biofilm formation, the supernatants were again removed, and the wells rinsed with PBS again. Once the wells were washed, 100 μl of a 0.5% crystal violet (CV) solution was added to all wells. After 20 min, the excess CV was removed by washing the plates under running tap water. Finally, bound CV was released by adding 150 μl of 33% acetic acid (Sigma). The absorbance was measured at 590 nm. All steps were carried out at room temperature.

### General synthesis of protected ligands 3 and 4Gal

The relevant peracetylated carbohydrate was treated with TMS-N_3_ and SnCl_4_ according to an established protocol to introduce azide functionality into the anomeric position.^49^ This carbohydrate azide (2 equiv.), CuI (0.5 equiv.), DIPEA (3 equiv.) and 2,6-bis(trimethylsilylethynyl)pyridine (1 equiv.) were suspended in DMF and heated under microwave irradiation at 100 °C for 20 minutes. The reaction mixture was poured into an aqueous solution of EDTA/NH_4_OH, extracted three times into CH_2_Cl_2_, washed with water and brine, dried over MgSO_4_, filtered and concentrated under reduced pressure; the product was purified by flash chromatography (CH_2_Cl_2_–CH_3_OH gradient) or trituration with cold CH_3_OH, yielding 3 or 4Gal as an off-white solid, which was used without further purification (3Gal and 3Glc were previously reported^32^).

#### 3Man

Synthesised according to the general procedure with azide 1Man (0.330 g, 0.88 mmol), CuI (0.042 g, 0.22 mmol), DIPEA (0.24 mL, 1.32 mmol) and 2,6-bis(trimethylsilylethynyl)pyridine (0.120 g, 0.44 mmol). Yield: 0.270 g, 0.31 mmol, 68%. Product decomposed above 200 °C. HRMS (*m*/*z*) (ESI+): calculated for C_37_H_43_N_7_O_18_Na^+^*m*/*z* = 896.2556 [M + Na]^+^. Found *m*/*z* = 896.2532; ^1^H NMR (CDCl_3_, 500 MHz): *δ* = 2.05–2.11 (m, 18H, 3 OAc CH_3_), 2.21 (s, 6H, OAc CH_3_), 3.93–4.00 (m, 2H, Man CH), 3.99–4.15 (m, 2H, Man C*H*H), 4.39 (dd, 2H, *J* = 12.6, 5.1 Hz, Man CH*H*), 5.42 (t, 2H, *J* = 8.9 Hz, Man CH), 5.98 (dd, 2H, *J* = 8.9, 3.5 Hz, Man CH), 6.02 (t, 2H, *J* = 3.5 Hz, Man CH), 6.10 (d, 2H, *J* = 2.5 Hz, anomeric Man CH), 7.93 (t, 1H, *J* = 7.8 Hz, pyr CH), 8.16 (d, 2H, *J* = 7.8 Hz, pyr CH), 8.37 (s, 2H, triazolyl CH); ^13^C NMR (CDCl_3_, 101 MHz): *δ* = 20.6, 20.65, 20.66, 20.71 (4 OAc CH_3_), 61.5 (Man CH_2_), 66.0, 68.3, 68.7, 72.3 (4 Man CH), 83.8 (Man anomeric CH), 120.0 (pyr CH), 122.4 (triazolyl CH), 138.0 (pyr CH), 148.7 (qt), 149.4 (qt), 169.3, 169.6, 169.7, 170.4; FT-IR (ATR, cm^−1^): 2981, 2258, 1744 (s), 1611, 1576, 1430, 1369, 1214 (s), 1130, 1051, 1028, 907, 808, 727.

#### 3Lac

Yield: 0.210 g, 0.145, 63%. Product decomposed above 200 °C. HRMS (*m*/*z*) (MALDI+): calculated for C_61_H_75_N_7_O_34_Na^+^*m*/*z* = 1472.4253. Found *m*/*z* = 1472.4188; ^1^H NMR (CDCl_3_, 400 MHz): *δ* = 1.85 (s, 6H, OAc CH_3_), 1.94 (s, 6H, OAc CH_3_), 1.98–2.09 (m, 24H, OAc CH_3_), 2.13 (s, 6H, OAc CH_3_), 4.01–4.25 (m, 6H, 3 Lac CH), 3.85–4.02 (m, 6H, Lac CH_2_ and Lac C*H*H), 4.46 (app d, 2H, Lac CH*H*), 4.54 (d, 2H, *J* = 7.8 Hz, anomeric Gal CH), 4.97 (dd, 2H, *J* = 10.4, 3.3 Hz, Lac CH), 5.15–5.04 (m, 2H, Lac CH), 5.34 (d, 2H, *J* = 2.8 Hz, Lac CH), 5.42 (dd, 2H, *J* = 17.1, 8.7 Hz, Lac CH), 5.52 (t, 2H, *J* = 9.4 Hz, Lac CH), 5.89 (d, 1H, *J* = 9.3 Hz, anomeric Glc CH), 7.83 (t, 1H, *J* = 7.8 Hz, pyr CH), 8.04 (d, 2H, *J* = 7.8 Hz, pyr CH), 8.32 (s, 2H, triazolyl CH); ^13^C NMR (CDCl_3_, 101 MHz): *δ* = 20.2, 20.5, 20.58, 20.58, 20.6, 20.70, 20.73 (7 Ac CH_3_), 60.7 (Lac CH_2_), 61.9 (Lac CH_2_), 66.5, 69.0, 70.6, 70.8, 70.9, 72.8, 75.7, 75.9 (8 Lac CH), 85.6 (anomeric Glc CH), 101.1 (anomeric Gal CH), 119.8 (pyr CH), 120.8 (triazolyl CH), 137.7 (pyr CH), 148.7 (qt), 149.4 (qt), 169.0, 169.2, 169.5, 169.98, 170.03, 170.2, 170.3 (7 Ac C=O qt); FT-IR (ATR, cm^−1^): 2943, 1743 (s), 1612, 1576, 1435, 1368, 1212 (s), 1045, 915, 810, 732.

#### 4Gal

Synthesised according to the general procedure from azide 2Gal (0.152 g, 0.30 mmol), yielding the desired product as a brown oil. Concentration *in vacuo* yielded a very hygroscopic off-white solid. Yield: 0.135 g, 0.12 mmol, 79%. HRMS (*m*/*z*) (ESI+): calculated for C_49_H_67_N_7_O_24_Na^+^*m*/*z* = 1160.4130 [M + Na]^+^. Found *m*/*z* = 1160.4137; ^1^H NMR (CDCl_3_, 500 MHz): *δ* = 1.98 (s, 6H, Ac CH_3_), 2.02 (s, 6H, Ac CH_3_), 2.04 (s, 6H, Ac CH_3_), 2.14 (s, 6H, Ac CH_3_),3.56–3.72 (m, 14H, 3 × CH_2_ and Gal C^6^H*H*), 3.85–3.95 (m, 4H, Gal C^6^*H*H and Gal CH), 3.98 (t, 4H, *J* = 5.3 Hz, CH_2_), 4.07–4.20 (m, 4H, CH_2_), 4.48 (d, 2H, *J* = 8.0 Hz, Gal anomeric CH), 4.40–4.69 (m, 4H, CH_2_), 5.00 (dd, 2H, *J* = 3.4, 10.5 Hz, Gal C^3^H), 5.18 (dd, 2H, *J* = 8.0, 10.5 Hz, Gal C^2^H), 5.38 (d, 2H, *J* = 2.6 Hz, Gal CH), 7.85–7.91 (m, 1H, 4-pyr CH), 8.09 (d, 2H, *J* = 7.8 Hz, 3- and 5-pyr CH), 8.31 (s, 2H, triazolyl CH); ^13^C NMR (CDCl_3_, 126 MHz): *δ* = 20.57, 20.65, 20.67, 20.7 (4 × OAc CH_3_), 50.5 (CH_2_), 61.2 (CH_2_), 67.0 (Gal CH), 68.8 (Gal CH), 69.1 (Gal C^6^H_2_), 69.5 (CH_2_), 70.2 (CH_2_), 70.4 (CH_2_), 70.61 (CH_2_), 70.62 (Gal CH), 70.8 (Gal CH), 101.3 (Gal anomeric CH), 119.3 (3- and 5-pyr CH), 123.3 (triazolyl CH), 137.7 (4-pyr CH), 148.2 (qt), 150.1 (qt), 169.4, 170.1, 170.2, 170.4 (4 OAc qt C=O); FT-IR (ATR, cm^−1^): 2884, 1742 (s), 1608, 1575, 1435, 136, 1216 (s), 1174, 1133, 1042 (s), 915, 813, 730.

### General synthesis of deprotected ligands 5 and 6

To a suspension of the relevant protected ligand 3 or 4 in CH_3_OH (10 mL) was added a 1 M solution of NaOCH_3_ in CH_3_OH (0.1 mL). The reaction mixture was stirred at room temperature. Upon completion, the solution was neutralised by addition of DOWEX® 50X8 H^+^ resin, filtered and concentrated under reduced pressure, yielding the free sugar ligands as off-white solids (5Glc and 5Gal), or amber oils (5Man, 5Lac and 6Gal).

#### 5Gal

Synthesised according to the general procedure from 3Gal (0.090 g, 0.10 mmol) and 1 M NaOCH_3_ solution (0.1 mL). Yield: 0.035 g, 0.065 mmol, 63%. m.p. 238–246 °C. Anal. calcd for C_21_H_27_N_7_O_10_·2.5(H_2_O) (582.524 g mol^−1^), C 43.29, H 5.53, N 16.83%; found C 42.90, H 5.04, 16.38%. HRMS (*m*/*z*) (ESI−): calculated for C_21_H_26_N_7_O_10_^−^*m*/*z* = 536.1741 [M−H]^−^. Found *m*/*z* = 536.1740; calculated for C_21_H_27_N_7_O_10_Cl^−^*m*/*z* = 572.1507 [M + Cl]^−^. Found *m*/*z* = 572.1526; ^1^H NMR (D_2_O, 500 MHz): *δ* = 3.63–3.79 (m, 4H, Gal CH_2_), 3.82 (dd, 2H, *J* = 9.8, 3.3 Hz, Gal CH), 3.92–3.97 (m, 2H, Gal CH), 4.02 (d, 2H, *J* = 3.3 Hz, Gal CH), 4.20 (t, 2H, *J* = 9.5 Hz, Gal C^2^H), 5.67 (d, 2H, *J* = 9.2 Hz, anomeric CH), 7.69 (d, 2H, *J* = 7.8 Hz, pyr CH), 7.82 (t, 1H, *J* = 7.8 Hz, pyr CH), 8.60 (s, 2H, triazolyl CH); ^13^C NMR (D_2_O, 126 MHz): *δ* = 60.9 (Gal CH_2_), 68.6, 69.8, 72.9, 78.4 (4 Gal CH), 88.2 (anomeric Gal CH), 120.5 (pyr CH), 123.2 (triazolyl CH), 139.2 (pyr CH), 147.1 (qt), 148.3 (qt); FT-IR (ATR, cm^−1^): 3288, 2895, 2115, 1647, 1577, 1389, 1318, 1230, 1086, 1049, 877, 809, 672, 610, 572, 561.

#### 5Glc

Yield: 0.050 g, 0.09 mmol, 78%. m.p. 242–248 °C. Anal. calcd for C_21_H_27_N_7_O_10_·2.5(H_2_O) (582.524 g mol^−1^), C 43.29, H 5.53, N 16.83%; found C 43.56, H 5.05, 16.63%. HRMS (*m*/*z*) (ESI+): calculated for C_21_H_28_N_7_O_10_^+^*m*/*z* = 538.1898 [M + H]^+^. Found *m*/*z* = 538.1902; calculated for C_21_H_27_N_7_O_10_Na^+^*m*/*z* = 560.1717 [M + Na]^+^. Found *m*/*z* = 560.1710; ^1^H NMR (CD_3_OD, 500 MHz): *δ* = 3.49–3.67 (m, 6H, CH_2_ and Glc CH), 3.75 (dd, 2H, *J* = 12.3, 5.5 Hz, Glc CH), 3.84–4.00 (m, 4H, Glc CH), 5.70 (d, 2H, anomeric Glc CH, *J* = 9.1 Hz), 7.98 (app br s, 3H, pyr CH), 8.86 (s, 2H, triazolyl CH); ^13^C NMR (D_2_O, 126 MHz): *δ* = 60.5 (Glc CH_2_), 68.9, 72.4, 75.8, 78.9 (4 Glc CH), 87.6 (anomeric Glc CH), 120.3 (pyr CH), 123.3 (triazolyl CH), 139.0 (pyr CH), 146.9 (qt), 148.2 (qt); FT-IR (ATR, cm^−1^): 3266, 2883, 1611, 1577, 1437, 1313, 1227, 1095, 1073, 1036, 899, 807, 675, 600, 577, 562.

#### 5Man

Yield: 0.012 g, 0.022 mmol, 69%. HRMS (*m*/*z*) (ESI+): calculated for C_21_H_27_N_7_O_10_Na^+^*m*/*z* = 560.1717 [M + Na]^+^. Found *m*/*z* = 560.1689; ^1^H NMR (D_2_O, 500 MHz): *δ* = 3.31–3.45 (m, 2H, Man CH), 3.61–3.80 (m, 6H, Man CH and Man CH_2_), 4.10 (dd, 2H, *J* = 3.5, 9.0 Hz, Man CH), 4.77 (dd, 2H, *J* = 3.5, 2.5 Hz, Man CH), 6.12 (d, 2H, *J* = 2.6 Hz, anomeric Man CH), 7.87 (d, 2H, *J* = 7.8 Hz, pyr CH), 7.96 (t, 1H, *J* = 7.8 Hz, pyr CH), 8.64 (s, 2H, triazolyl CH); ^13^C NMR (D_2_O, 126 MHz): *δ* = 60.4 (Man CH_2_), 66.5, 68.3, 70.5, 76.2 (4 Man CH), 86.9 (anomeric Man CH), 120.7 (pyr CH), 123.8 (triazolyl CH), 139.3 (pyr CH), 147.2 (qt), 148.6 (qt); FT-IR (ATR, cm^−1^): 3306 (br), 2457, 1577, 1438, 1215, 1033 (s), 794.

#### 5Lac

Yield: 0.085 g, 0.10 mmol, 77%. m.p. 207–212 °C. HRMS (*m*/*z*) (ESI+): calculated for C_33_H_47_N_7_O_20_Na^+^*m*/*z* = 884.2774 [M + Na]^+^. Found *m*/*z* = 884.2783; ^1^H NMR (D_2_O, 400 MHz): *δ* = 3.44–3.52 (m, 2H, Lac CH), 3.57 (dd, 2H, *J* = 10.0, 3.2 Hz, Lac CH), 3.62–3.86 (m, 16H, 5 Lac CH, Lac CH_2_, Lac C*H*H), 3.93 (m, 4H, Lac CH and Lac CH*H*), 4.40 (d, 2H, *J* = 7.6 Hz, anomeric Gal CH), 5.67 (d, 2H, *J* = 9.4 Hz, anomeric Glc CH), 7.42 (d, 2H, *J* = 7.3 Hz, pyr CH), 7.56–7.63 (m, 1H, pyr CH), 8.38 (s, 2H, triazolyl CH); ^13^C NMR (D_2_O, 101 MHz): *δ* = 59.9 (Lac CH_2_), 61.0 (Lac CH_2_), 68.5, 70.9, 72.1, 72.5, 74.5, 75.4, 77.4, 77.7 (8 Lac CH), 87.4 (anomeric Glc CH), 102.9 (anomeric Gal CH), 120.1 (pyr CH), 123.3 (triazolyl CH), 138.8 (pyr CH), 146.8 (qt), 147.9 (qt); FT-IR (ATR, cm^−1^): 3326 (br), 2885, 1610, 1578, 1375, 1231, 1032 (s), 894, 806.

#### 6Gal

Yield: 0.023 g, 0.023 mmol, 66%. HRMS (*m*/*z*) (ESI+): calculated for C_33_H_51_N_7_O_16_Na^+^*m*/*z* = 824.3290 [M + Na]^+^. Found *m*/*z* = 824.3248; ^1^H NMR (CD_3_OD, 500 MHz): *δ* = 3.41–3.48 (m, 4H, 2 × Gal CH), 3.49–3.54 (m, 2H, Gal CH), 3.59–3.69 (m, 14H, 3 × CH_2_ and Gal C^6^H*H*), 3.71 (t, 4H, *J* = 5.6 Hz, CH_2_), 3.90–3.96 (m, 2H, Gal CH), 4.00 (t, 4H, *J* = 5.0 Hz, CH_2_), 4.17 (d, 2H, *J* = 7.64 Hz, anomeric Gal CH), 4.68–4.73 (m, 4H, CH_2_), 7.95–8.01 (m, 3H, 4-pyr CH and triazolyl CH), 8.64–8.69 (m, 2H, 3- and 5-pyr CH); ^13^C NMR (CD_3_OD, 126 Mz): *δ* = 50.3 (CH_2_), 61.1 (CH_2_), 68.2 (Gal C^6^H_2_), 68.8 (CH_2_), 68.9 (Gal CH), 70.01 (CH_2_), 70.05 (CH_2_), 71.1 (Gal CH), 73.5 (Gal CH), 75.2 (Gal CH), 103.6 (anomeric Gal CH), 118.7 (pyr CH), 124.2 (pyr CH), 137.9 (triazolyl CH), 147.5 (qt), 149.9 (qt); FT-IR (ATR, cm^−1^): 3367 (br), 2885, 2496, 1612, 1350, 1035 (s), 808, 776.

### Preparation of Ru(ii) complexes of btp ligands

Carbohydrate ligands, 5 or 6 (2 equiv.) were dissolved in 7 mL 70% aqueous ethanol solution and RuCl_3_·3H_2_O (1 equiv.) added. The reaction mixture was heated under microwave irradiation to 120 °C for 40 minutes and concentrated under reduced pressure to yield the product as very hygroscopic orange or red solids 7 or 8.

#### 7Gal

Synthesised according to the general procedures from 5Gal (0.020 g, 0.037 mmol) and RuCl_3_·3H_2_O (0.005, 0.021 mmol). Residue was dissolved in CH_3_OH and filtered, concentrating the filtrate to give the product as a hygroscopic red solid. Yield: 0.018 g, 0.014 mmol, 75%. Product decomposed above 180 °C. Anal. calcd for C_42_H_54_N_14_O_20_·10(H_2_O)·2NaCl (1544.821 g mol^−1^), C 32.66, H 4.82, N 12.69%; found C 32.81, H 4.33, 12.57%. HRMS (*m*/*z*) (ESI+): calculated for C_42_H_54_N_14_O_20_Ru^2+^*m*/*z* = 588.1341 [M−2Cl]^2+^. Found *m*/*z* = 588.1350; ^1^H NMR (D_2_O, 500 MHz): *δ* = 3.48–3.58 (m, 4H, 2 Gal CH), 3.63 (dd, 2H, *J* = 10.0, 3.0 Hz, Gal CH*H*), 3.68–3.77 (m, 2H, Gal C*H*H), 3.78–3.93 (m, 4H, 2 Gal CH), 5.36 (d, 2H, *J* = 9.2 Hz, anomeric Gal CH), 8.20 (app s, 3H, pyr CH), 8.97 (s, 2H, triazolyl CH); ^13^C NMR (D_2_O, 126 MHz): *δ* = 60.7 (Gal CH_2_), 68.3, 69.3, 72.5, 78.4 (4 Gal CH), 88.9 (anomeric Gal CH), 120.8 (pyr CH), 124.6 (triazolyl CH), 137.9 (pyr CH), 150.1 (qt), 150.4 (qt); FT-IR (ATR, cm^−1^): 3288 (s, br), 1622, 1389, 1209, 1089 (s), 887, 805.

#### 7Glc

Yield: 0.024 g, 0.013 mmol, 66%. Product decomposed above 180 °C. HRMS (*m*/*z*) (ESI+): calculated for C_42_H_54_N_14_O_20_Ru^2+^*m*/*z* = 588.1341 [M−2Cl]^2+^. Found *m*/*z* = 588.1335; ^1^H NMR (D_2_O, 500 MHz): *δ* = 3.35 (t, 2H, *J* = 9.3 Hz, Glc CH), 3.40–3.57 (m, 8H, 2 Glc CH and CH_2_), 3.58–3.70 (m, 4H, 2 Glc CH), 5.43 (d, 2H, *J* = 9.3 Hz, anomeric Glc CH), 8.20 (app s, 3H, pyr CH), 8.95 (s, 2H, triazolyl CH); ^13^C NMR (D_2_O, 126 MHz): *δ* = 62.7 (Glc CH_2_), 71.1, 74.4, 78.0, 81.4 (4 Glc CH), 90.8 (anomeric Glc CH), 123.4 (pyr CH), 127.3 (triazolyl CH), 151.2 (pyr CH), 152.7 (qt), 153.0 (qt); FT-IR (ATR, cm^−1^): 3286 (s, br), 2927, 1624, 1591, 1447, 1389 (s), 1335, 1263, 1208, 1095, 1051, 1015 (s), 895, 806, 611.

#### 7Man

Yield: 0.010 g, 0.008 mmol, 80%. HRMS (*m*/*z*) (ESI+): calculated for C_42_H_54_N_14_O_20_Ru^2+^*m*/*z* = 588.1341 [M−2Cl]^2+^. Found *m*/*z* = 588.1341; ^1^H NMR (D_2_O, 400 MHz); *δ* = 2.80–3.09 (m, 2H, Man CH), 3.38 (dd, 2H, *J* = 8.3, 3.1 Hz, Man CH*H*), 3.49–3.66 (m, 6H, 2 Man CH and Man C*H*H), 4.27 (br s, 2H, Man CH), 5.84 (d, 2H, *J* = 2.1 Hz, anomeric CH), 8.20 (m, 3H, pyr CH), 8.91 (s, 2H, triazolyl CH); ^13^C NMR (D_2_O, 101 MHz): *δ* = 60.1 (Man CH_2_), 66.1, 67.5, 70.2, 77.1 (4 Man CH), 87.6 (anomeric CH), 120.6, 120.8, 125.0, 150.0 (qt), 150.5 (qt); FT-IR (ATR, cm^−1^): 3264 (s, br), 1618, 1447, 1068, 1051, 799.

#### 7Lac

Yield: 0.011 g, 0.0058 mmol, 67%. HRMS (*m*/*z*) (ESI+): calculated for C_66_H_94_N_14_O_40_Ru^2+^*m*/*z* = 912.2402 [M−2Cl]^2+^. Found *m*/*z* = 912.2392; ^1^H NMR (D_2_O, 500 MHz): *δ* = 3.39 (dd, 2H, *J* = 9.9, 7.8 Hz, Lac CH), 3.51 (dd, 2H, *J* = 10.2, 3.2 Hz, Lac CH), 3.54–3.74 (m, 18H, 3 × Lac CH and 2 × Lac CH_2_), 3.75–3.85 (m, 4H, 2 × Lac CH), 4.29 (d, 2H, *J* = 7.8 Hz, anomeric Gal CH), 5.47 (d, 2H, *J* = 9.0 Hz, anomeric Glc CH), 8.17–8.21 (m, 3H, pyr CH), 8.96 (s, 2H, triazolyl CH); ^13^C NMR (D_2_O, 126 MHz) *δ* = 59.8 (Lac CH_2_) 61.0 (Lac CH_2_), 68.4, 70.8, 71.5, 72.4, 74.1, 75.3, 76.9, 77.7 (8 Lac CH), 88.1 (anomeric Glc CH, assigned by HSQC), 102.8 (anomeric Gal CH), 120.9 (pyr CH, assigned by HSQC), 137.9 (pyr CH, assigned by HSQC), 150.1 (qt), 150.4 (qt); FT-IR (ATR, cm^−1^): 3269 (s, br), 1627, 1400, 1027, 894.

#### 8Gal

Yield: 0.025 g, 0.014 mmol, 88%. Product decomposed above 180 °C. HRMS (*m*/z) (ESI+): calculated for C_66_H_102_N_14_O_32_Ru^2+^*m*/*z* = 852.2918 [M−2Cl]^2+^. Found *m*/*z* = 852.2890; ^1^H NMR (D_2_O, 600 MHz): *δ* = *δ* = 3.30–3.37 (m, 8H, 2 × CH_2_), 3.39 (dd, 2H, *J* = 10.1, 7.8 Hz, Gal CH), 3.43–3.46 (m, 4H, CH_2_), 3.50–3.56 (m, 4H, 2 × Gal CH), 3.57–3.69 (m, 10H, 2 × CH_2_, Gal CH*H*), 3.80 (app d, 2H, *J* = 4.1 Hz, Gal CH), 3.83–3.93 (m, 2H, Gal C*H*H), 4.25 (d, 2H, *J* = 7.8 Hz, anomeric Gal CH), 4.36 (br t, 4H, CH_2_), 8.17–8.21 (m, 3H, pyr CH), 8.80 (s, 2H, triazolyl CH); ^13^C NMR (D_2_O, 101 MHz): *δ* = 51.6 (CH_2_), 61.0 (CH_2_), 68.1 (CH_2_), 68.5 (CH_2_), 68.6 (Gal CH), 69.4 (CH_2_), 69.7 (Gal CH), 70.7 (Gal CH), 72.7 (Gal CH), 75.2 (Gal CH), 102.8 (anomeric Gal CH), 120.3 (pyr CH), 126.0 (pyr CH), 137.9 (triazolyl CH), 150.1 (qt), 150.5 (qt); FT-IR (ATR, cm^−1^): 3345 (s, br), 2923, 1622, 1404, 1060 (s), 809.

## Conflicts of interest

There are no conflicts to declare.

## Supplementary Material

RA-011-D0RA05107A-s001

RA-011-D0RA05107A-s002
