# Supplementary material for: Ruthenium-centred btp glycoclusters as inhibitors for Pseudomonas aeruginosa biofilm formation
Source: RSC Adv. 2021 May 4;11(27):16318–25. doi: 10.1039/d0ra05107a (PMC9030604; doi:10.1039/d0ra05107a)

Electronic Supplementary Material (ESI) for RSC Advances.
This journal is © The Royal Society of Chemistry 2021

Supporting Information for:

Ruthenium-centred btp glycoclusters as inhibitors for *Pseudomonas aeruginosa* biofilm formation

Ciaran O'Reilly,[a,b] Salvador Blasco,[a] Bina Parekh[b], Helen Collins[c], Gordon Cooke[b,c], Thorfinnur Gunnlaugsson,[a] and Joseph P. Byrne*[d]

[a.] *School of Chemistry and Trinity Biomedical Sciences Institute, Trinity College Dublin, Ireland.*
[b.] *School of Medicine, University College Dublin, Belfield, Dublin 4, Ireland*
[c.] *Department of Applied Science, Tallaght Campus, Technological University Dublin*
[d.] *School of Chemistry, National University of Ireland Galway, University Road, Galway, Ireland.*
* *joseph.byrne@nuigalway.ie*

Contents:

page

NMR Spectra S1
Biofilm Formation Assay for Ligand **6Gal** S15
Minimum Inhibitory Concentration (MIC) Testing S15
Minimum Bactericidal Concentration (MBC) Testing S17
HeLa Cytotoxicity Testing S18
Supplementary X-ray crystallography data S18

NMR spectra

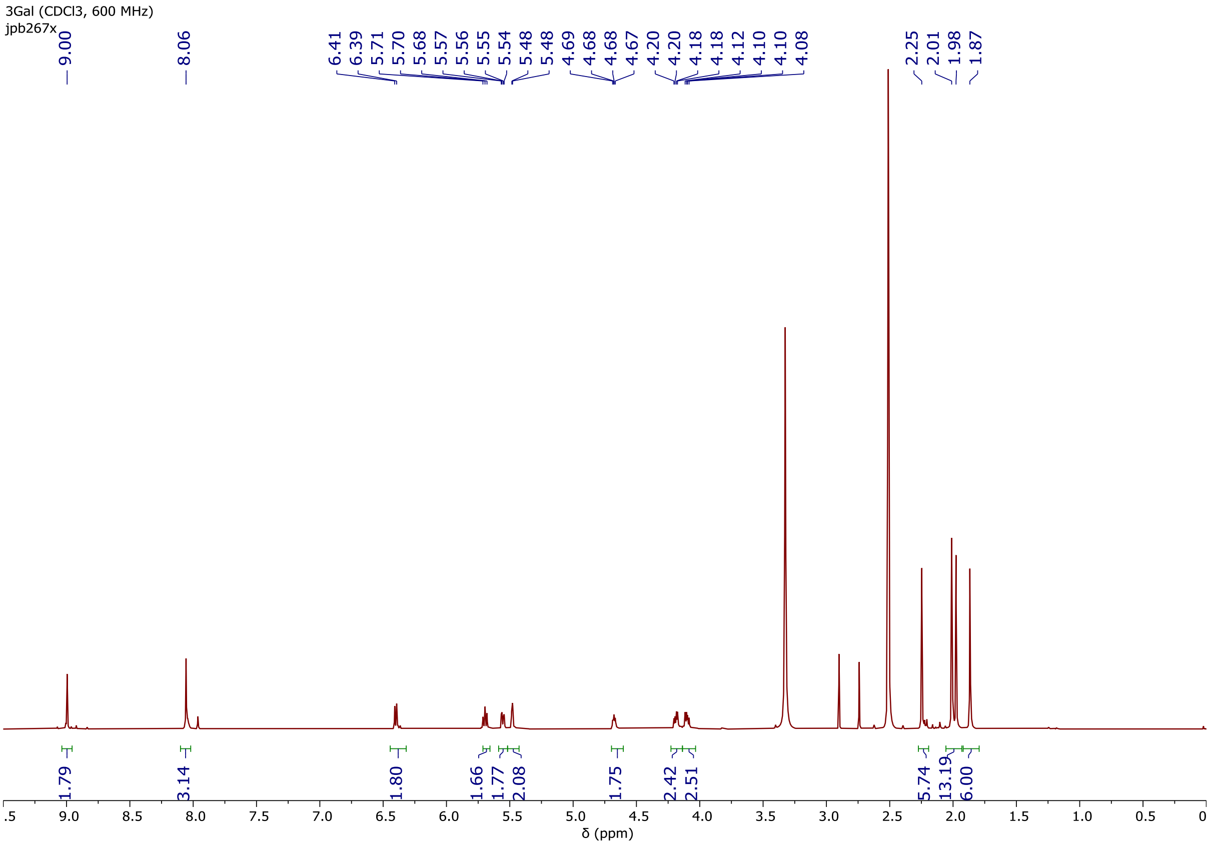

Figure S1. *^{1}H NMR spectrum ($CDCl_3$, 600 MHz) of* ***3Gal***

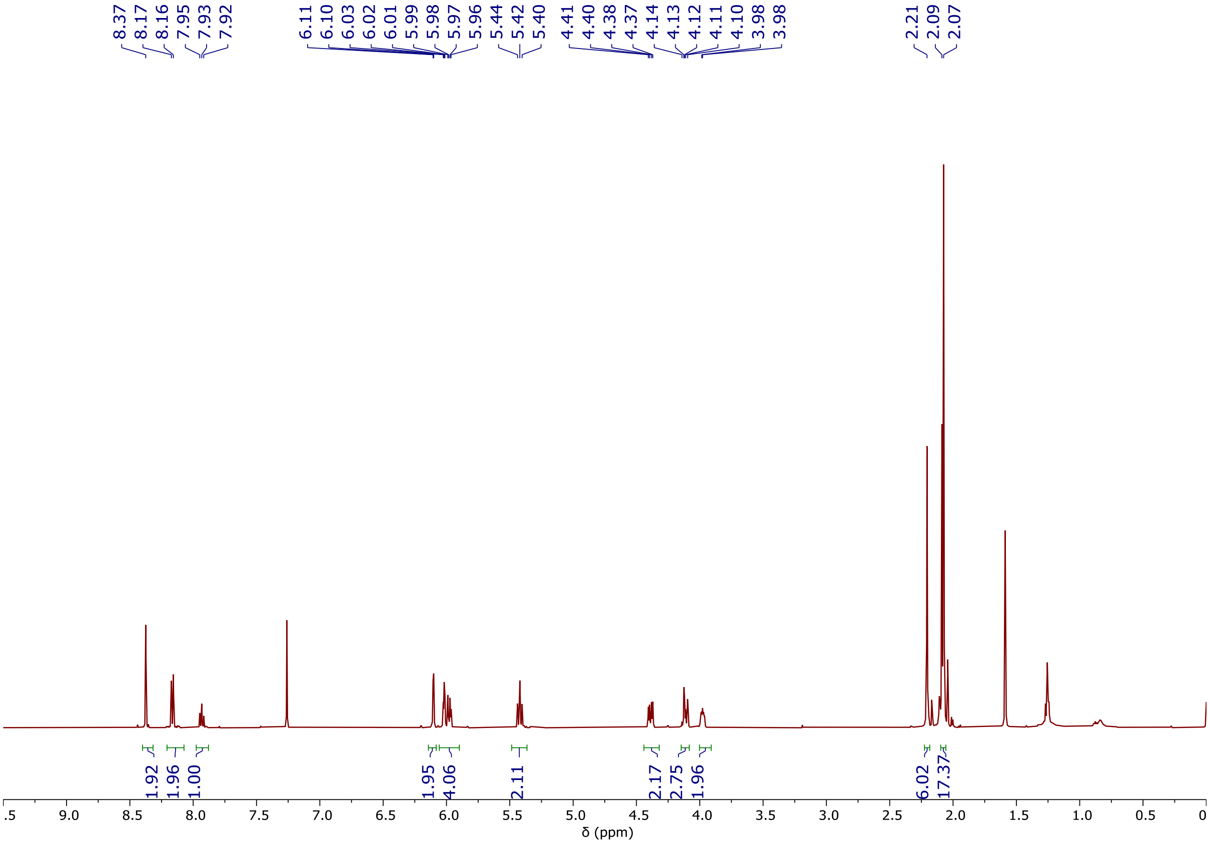

Figure S2. *^{1}H NMR spectrum ($CDCl_3$, 500 MHz) of* ***3Man***

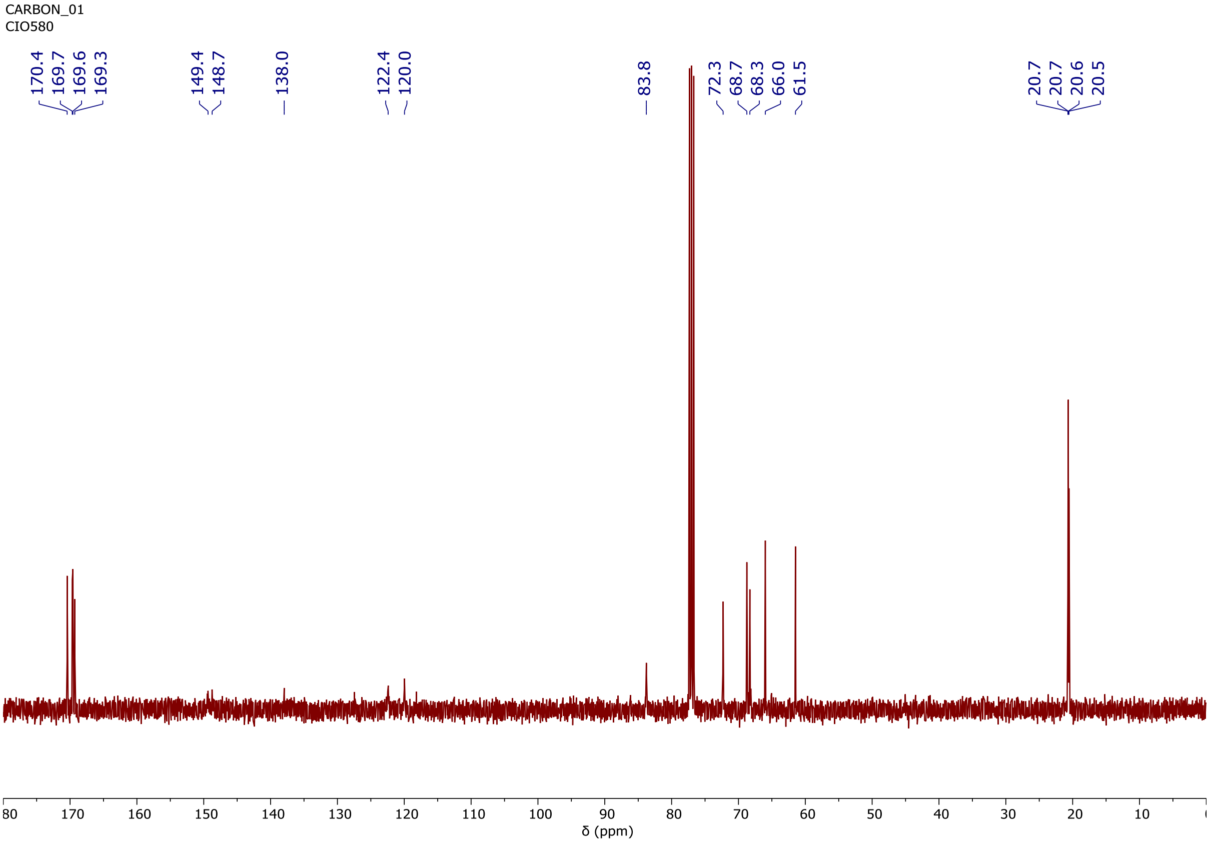

Figure S3. *^{13}C NMR spectrum ($CDCl_3$, 101 MHz) of* ***3Man***

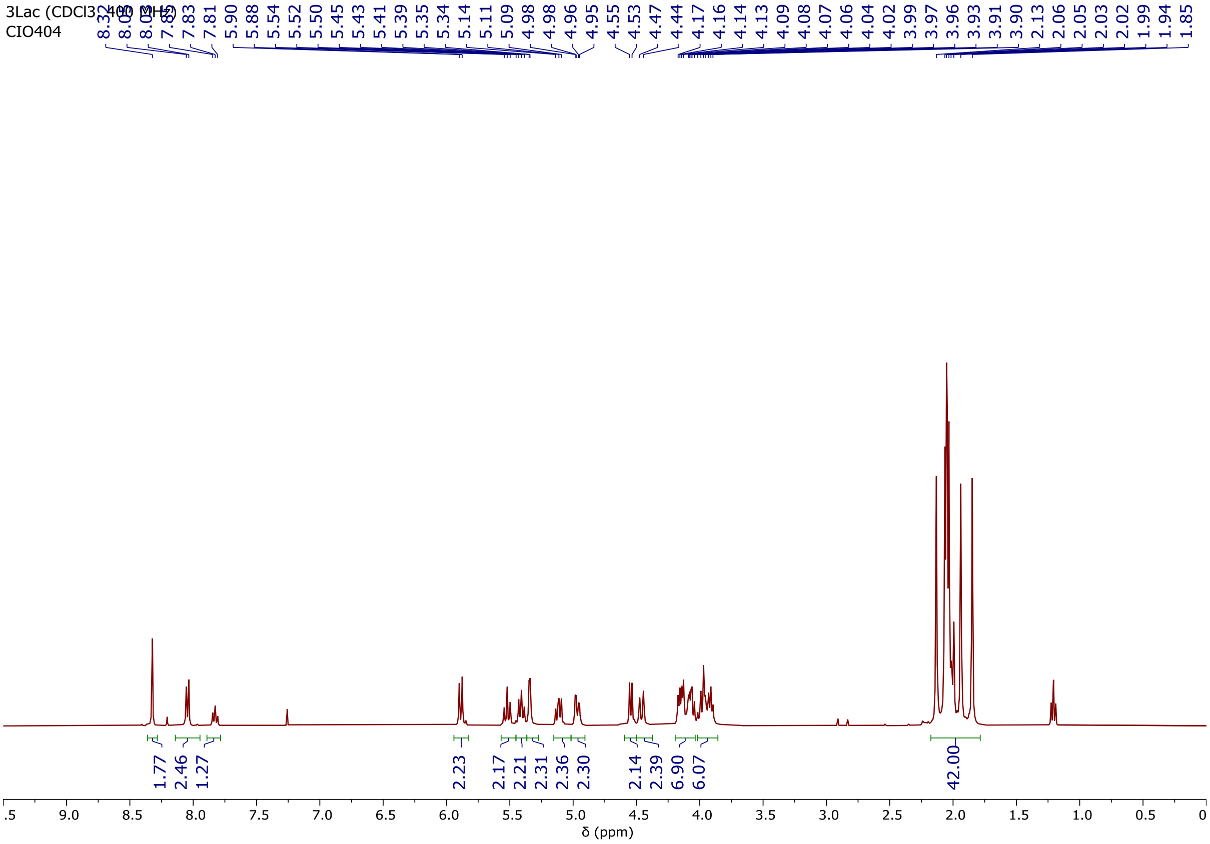

Figure S4. *^{1}H NMR spectrum ($CDCl_3$, 400 MHz) of **3Lac***

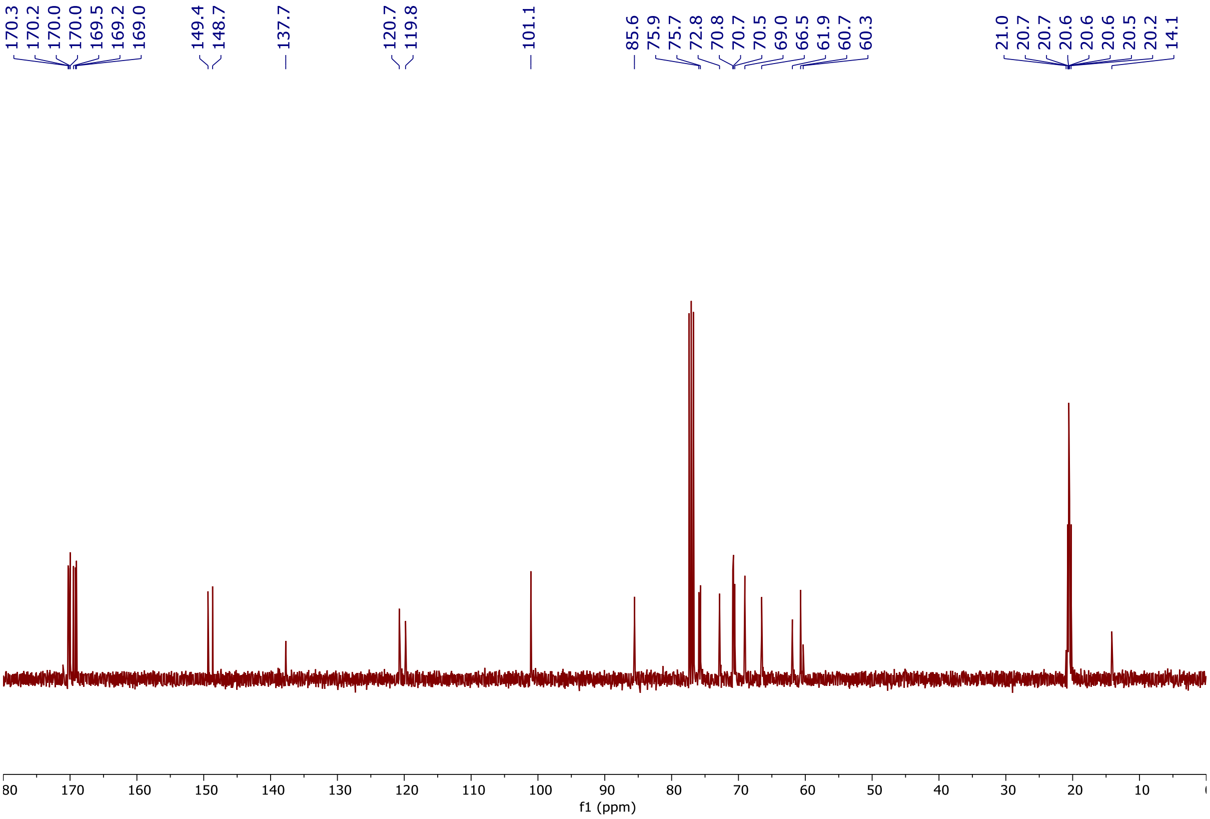

Figure S5. *^{13}C NMR spectrum ($CDCl_3$, 101 MHz) of **3Lac***

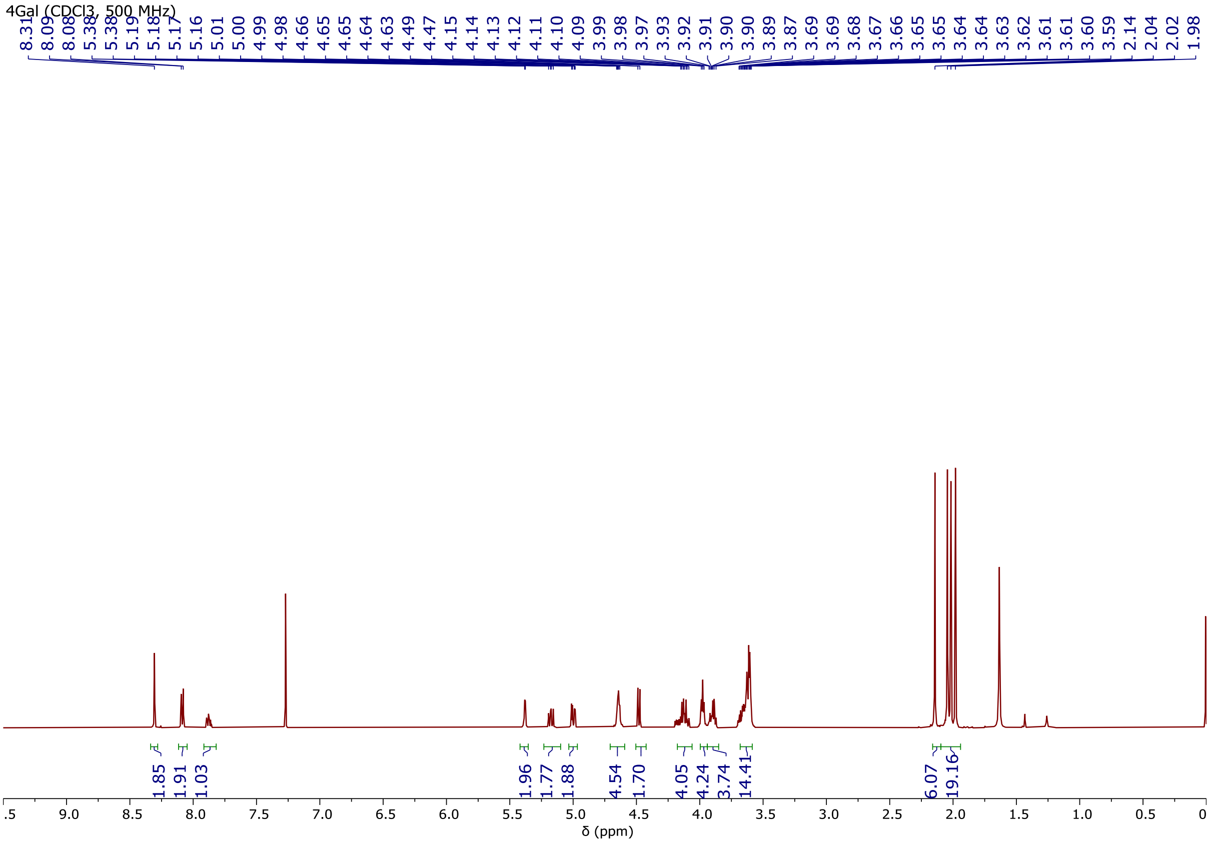

Figure S6. *^{1}H NMR spectrum ($CDCl_3$, 400 MHz) of **4Gal***

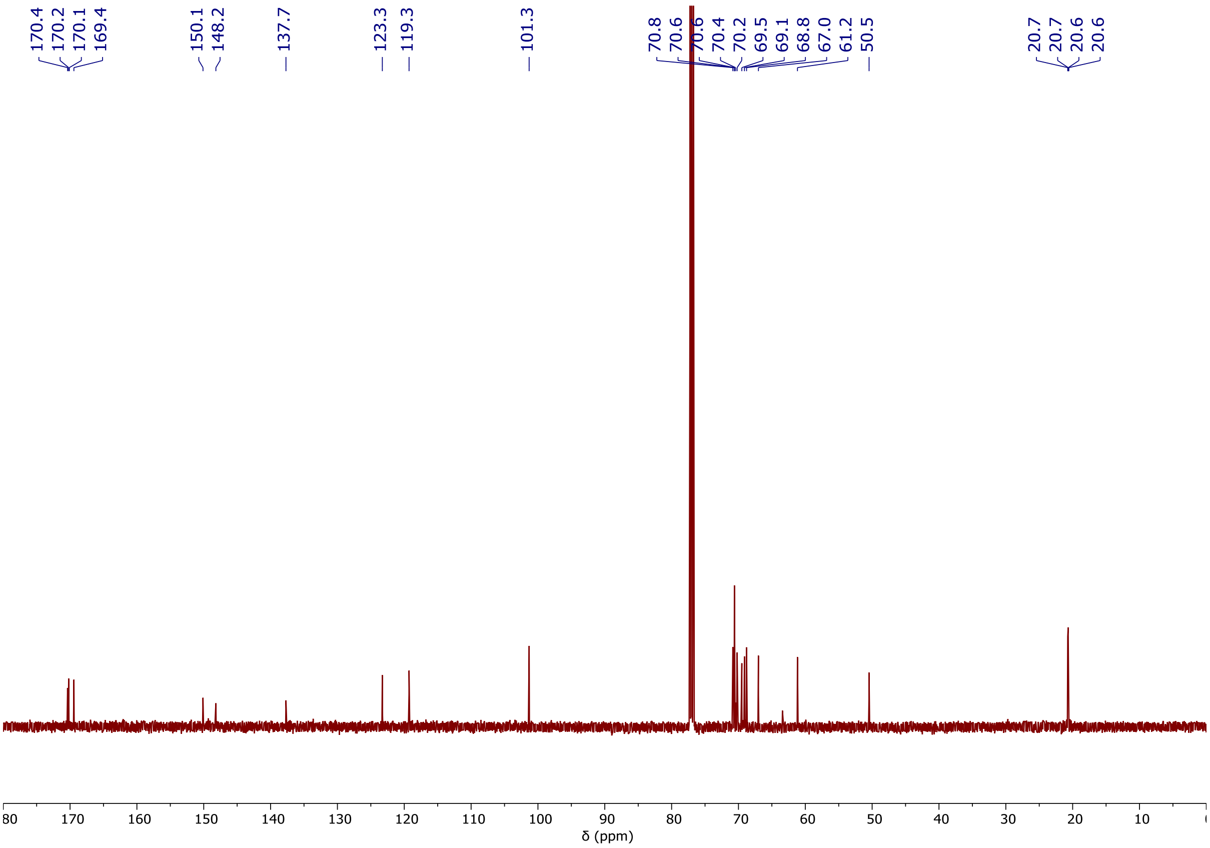

Figure S7. *^{13}C NMR spectrum ($CDCl_3$, 126 MHz) of **4Gal***

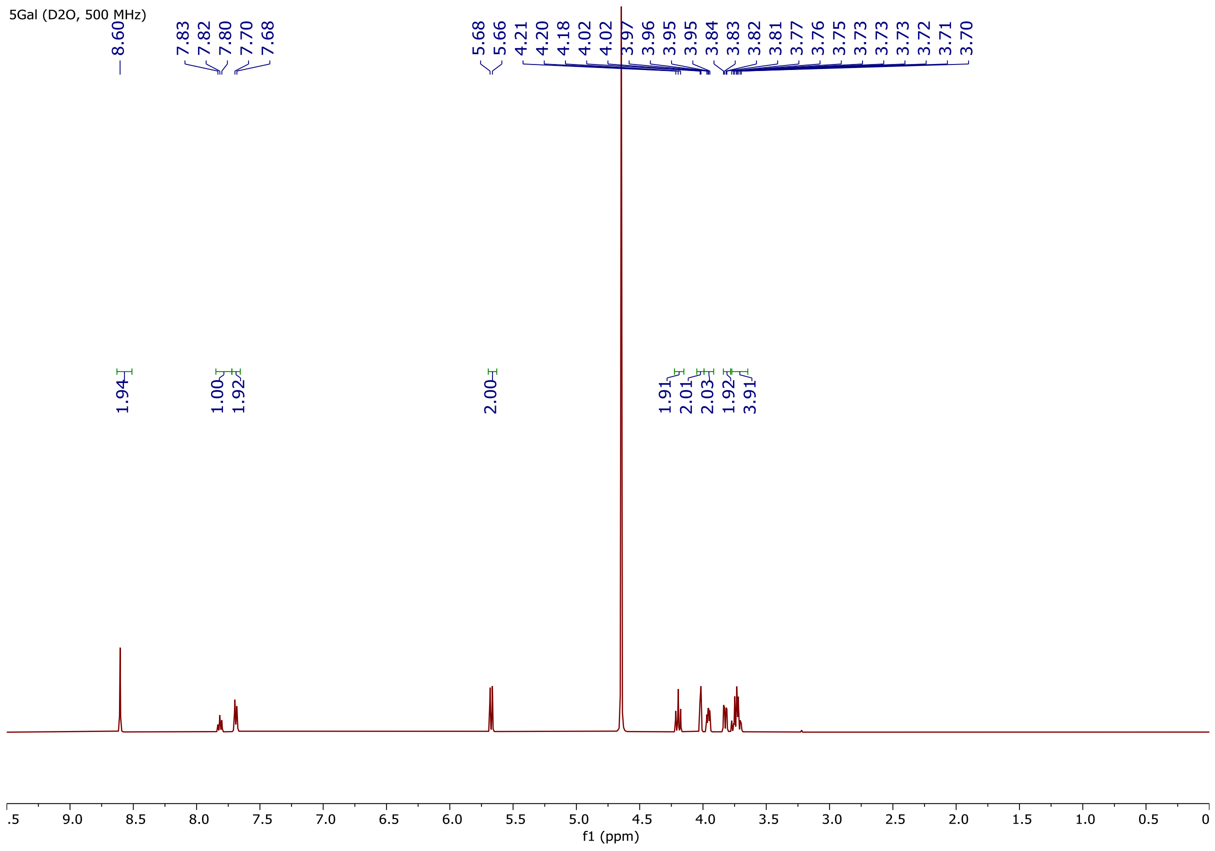

Figure S8. *^{1}H NMR spectrum (D_2O, 500 MHz) of* ***5Gal***

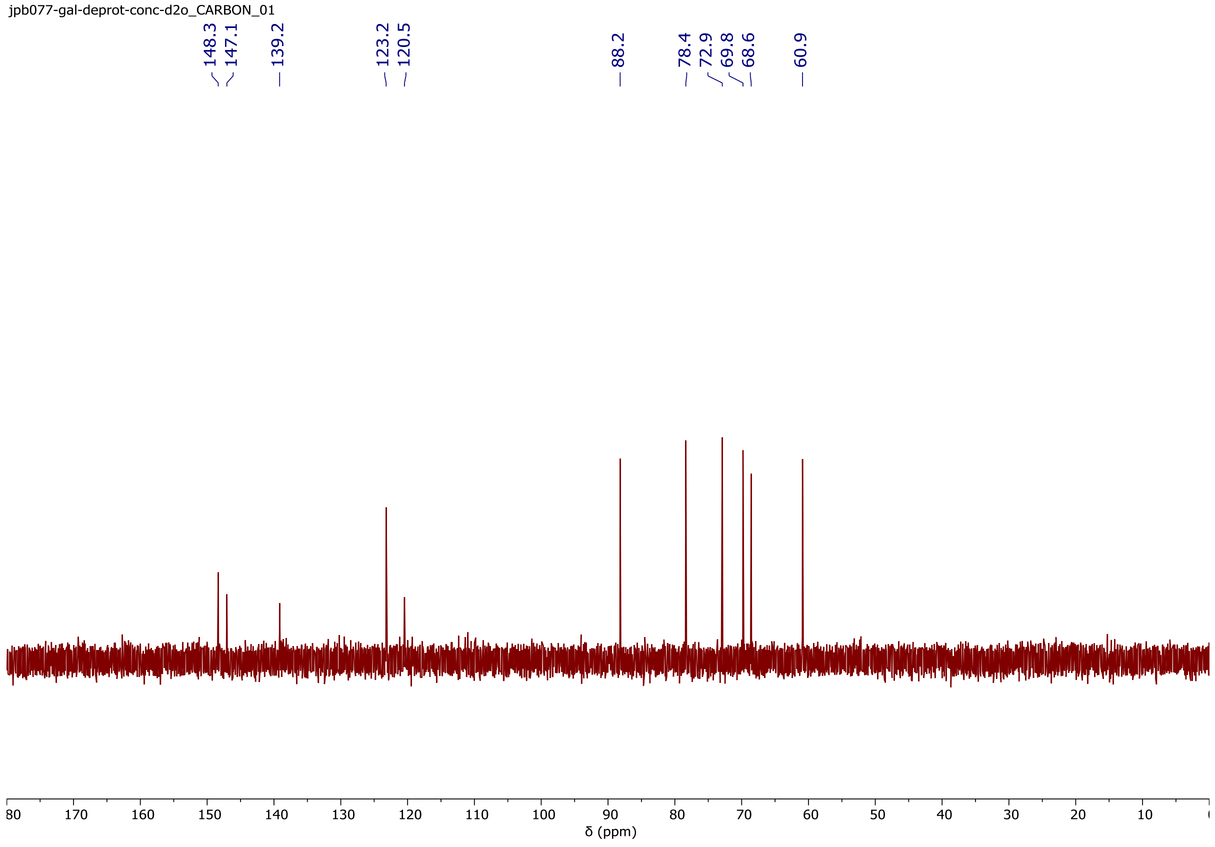

Figure S9. *^{13}C NMR spectrum (D_2O, 126 MHz) of* ***5Gal***

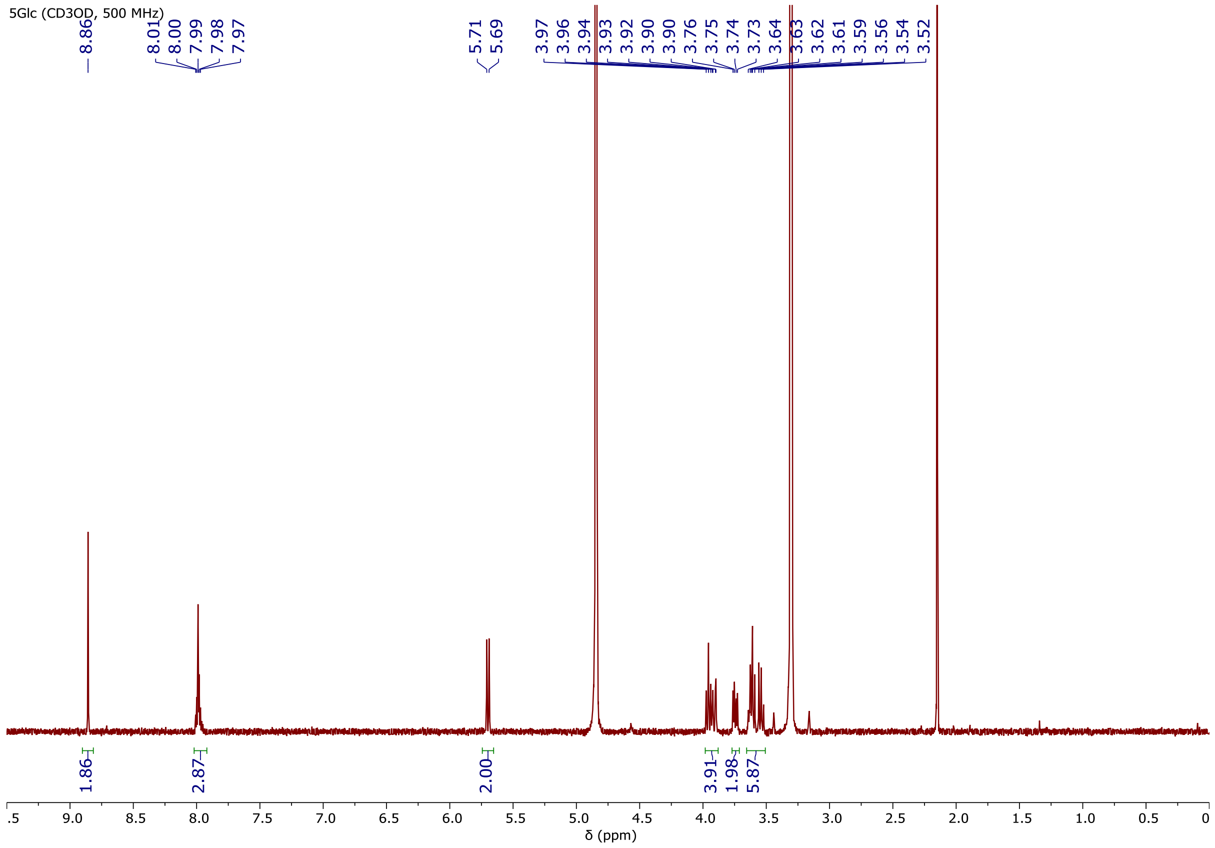

Figure S10. *^{1}H NMR spectrum (CD_3OD, 400 MHz) of* ***5Glc***

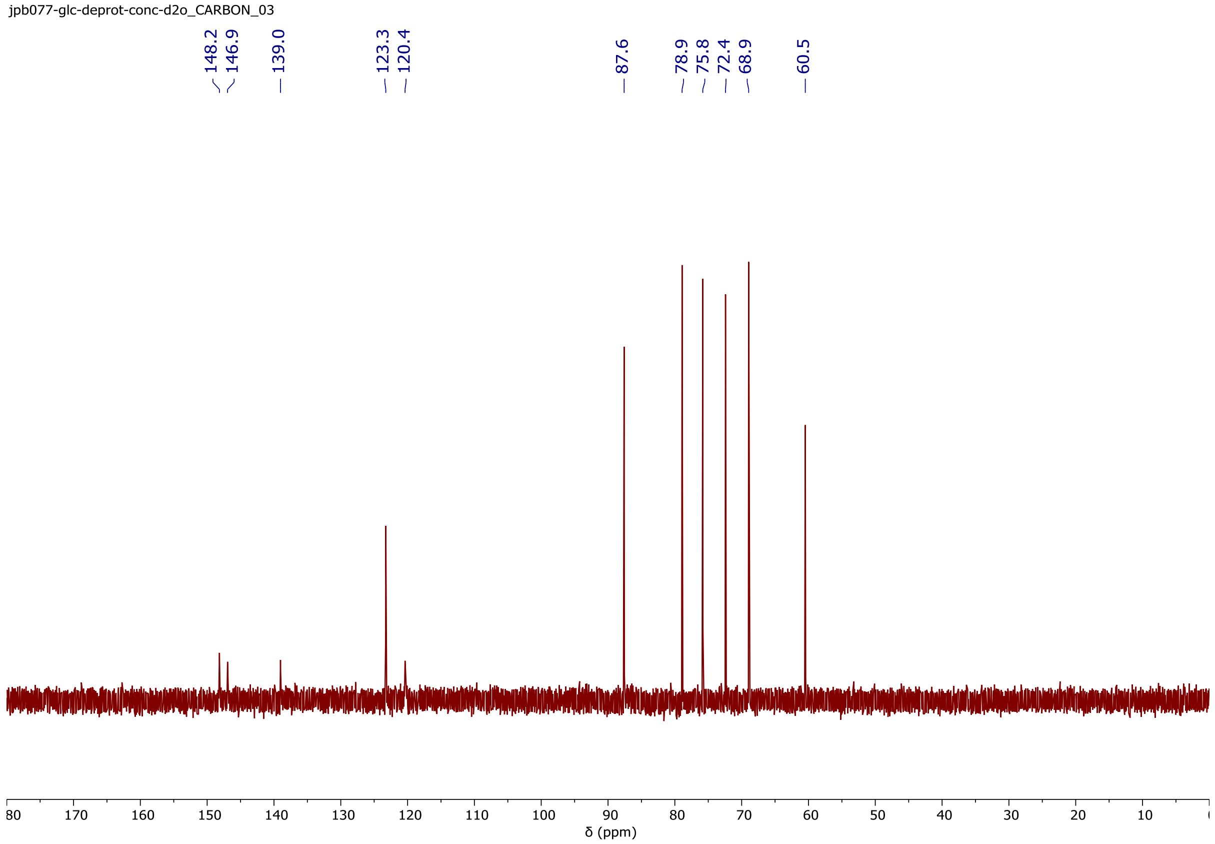

Figure S11. *^{13}C NMR spectrum (D_2O, 126 MHz) of* ***5Glc***

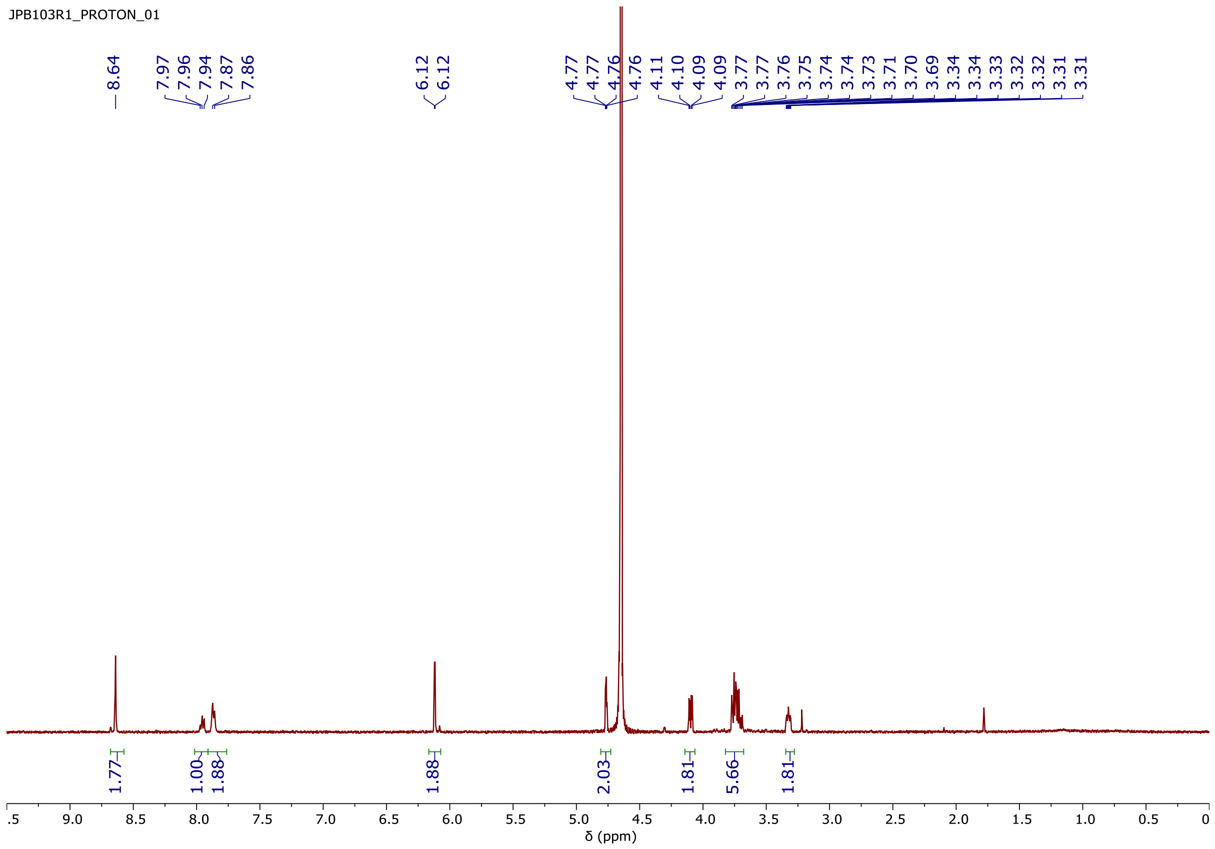

Figure S12. *¹H NMR spectrum (D₂O, 500 MHz) of* ***5Man***

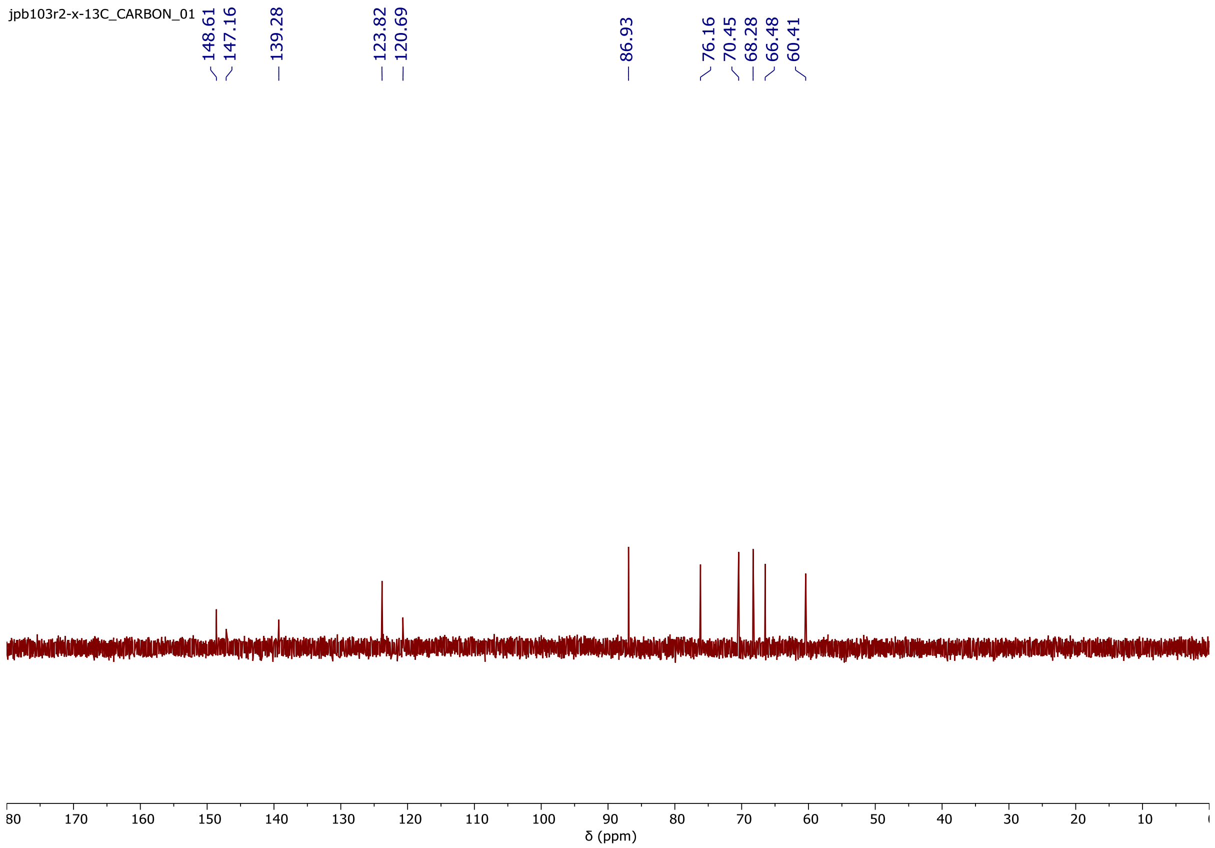

Figure S13. *¹³C NMR spectrum (D₂O, 126 MHz) of* ***5Man***

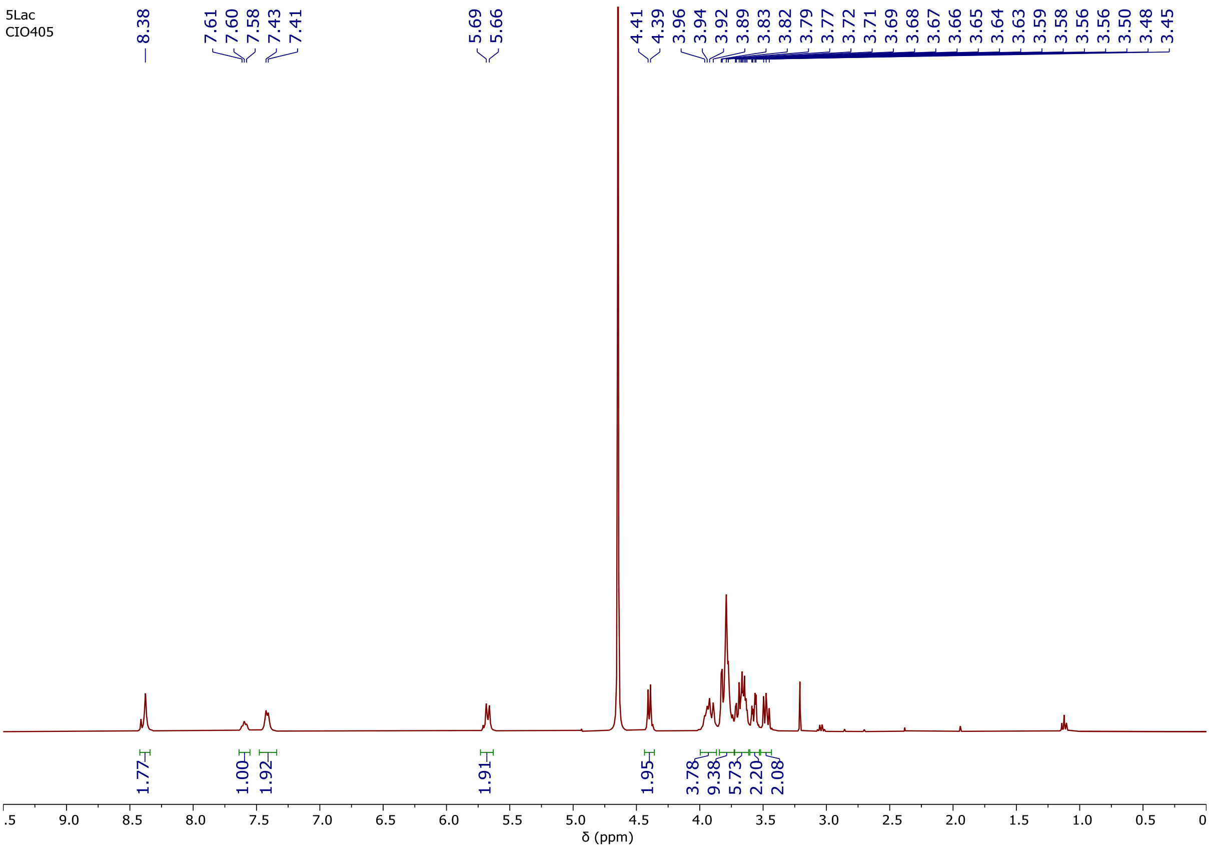

Figure S14. *¹H NMR spectrum (D_2O, 400 MHz) of* ***5Lac***

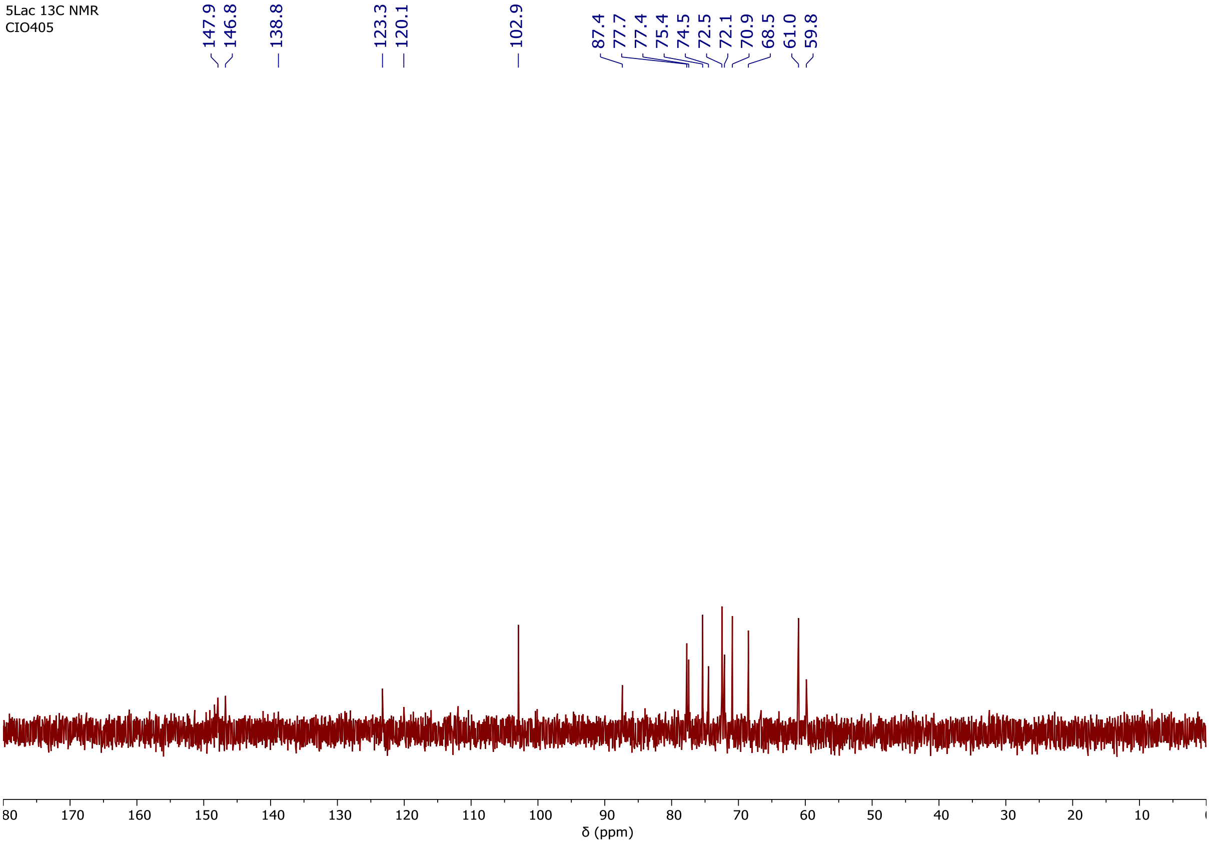

Figure S15. *¹³C NMR spectrum (D_2O, 101 MHz) of* ***5Lac***

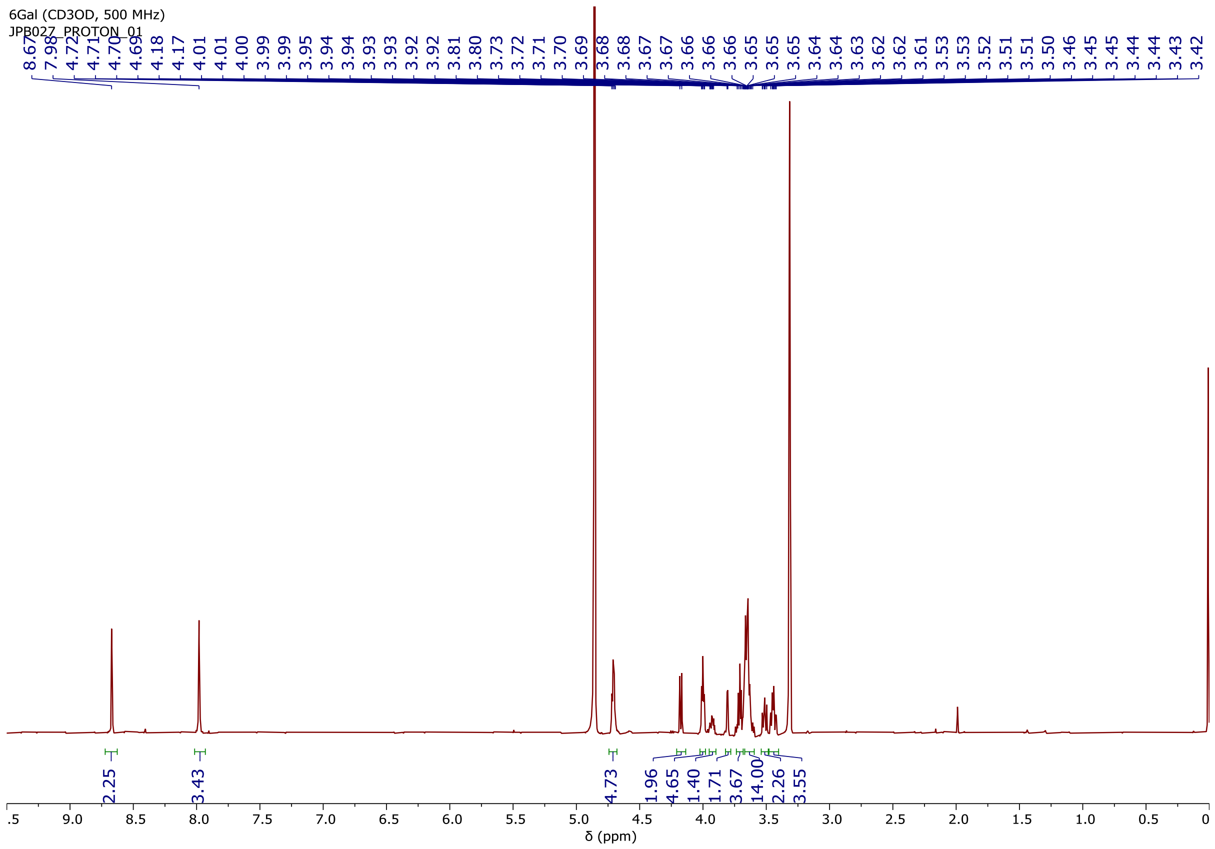

Figure S16. *^{1}H NMR spectrum (CD_3OD, 500 MHz) of **6Gal***

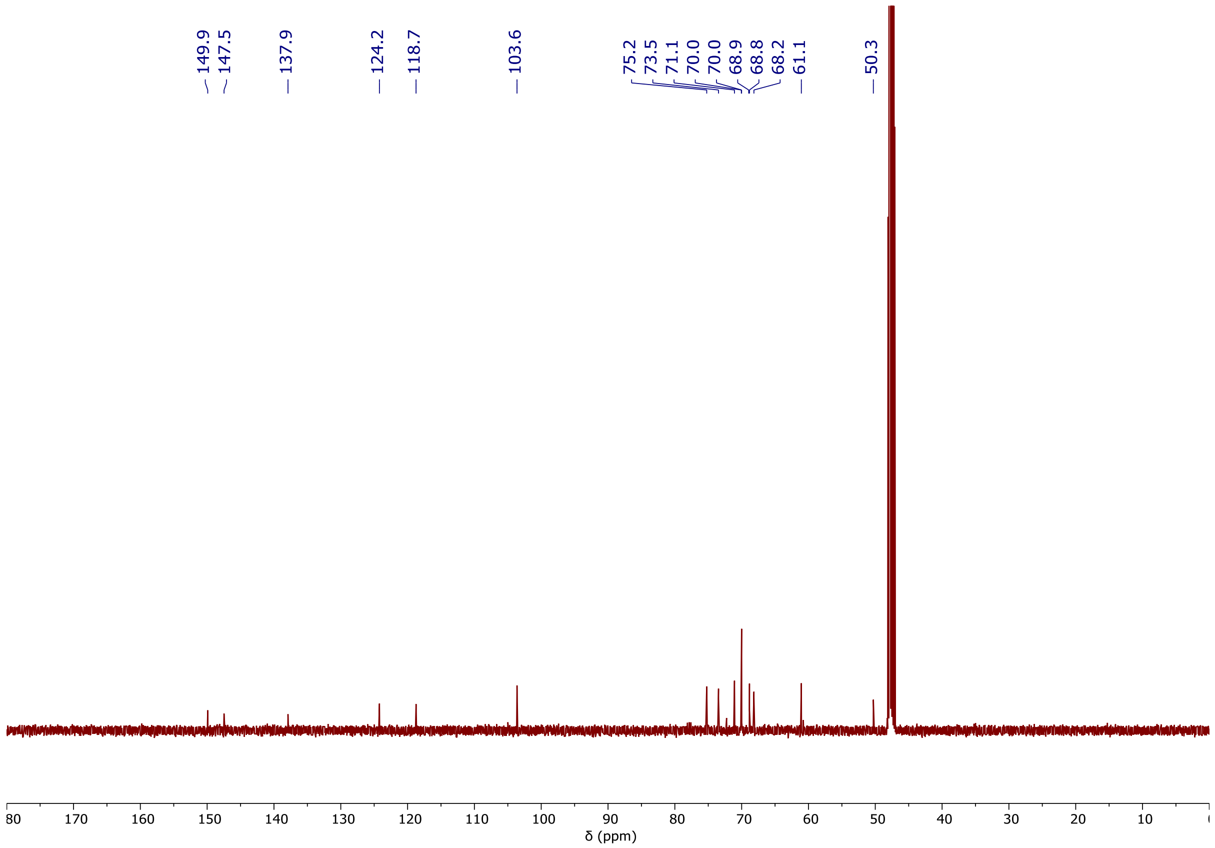

Figure S17. *^{13}C NMR spectrum (CD_3OD, 126 MHz) of **6Gal***

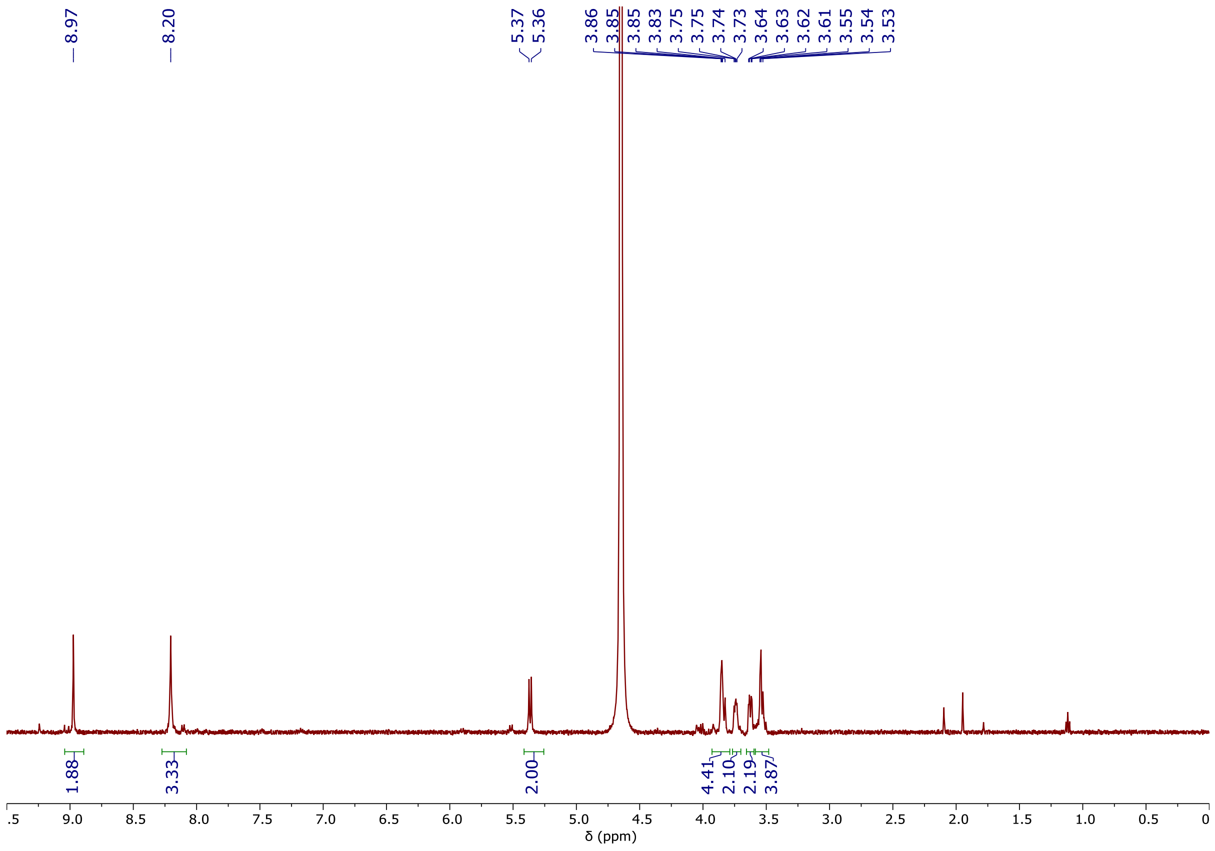

Figure S18. *^{1}H NMR spectrum (D_2O, 500 MHz) of **7Gal***

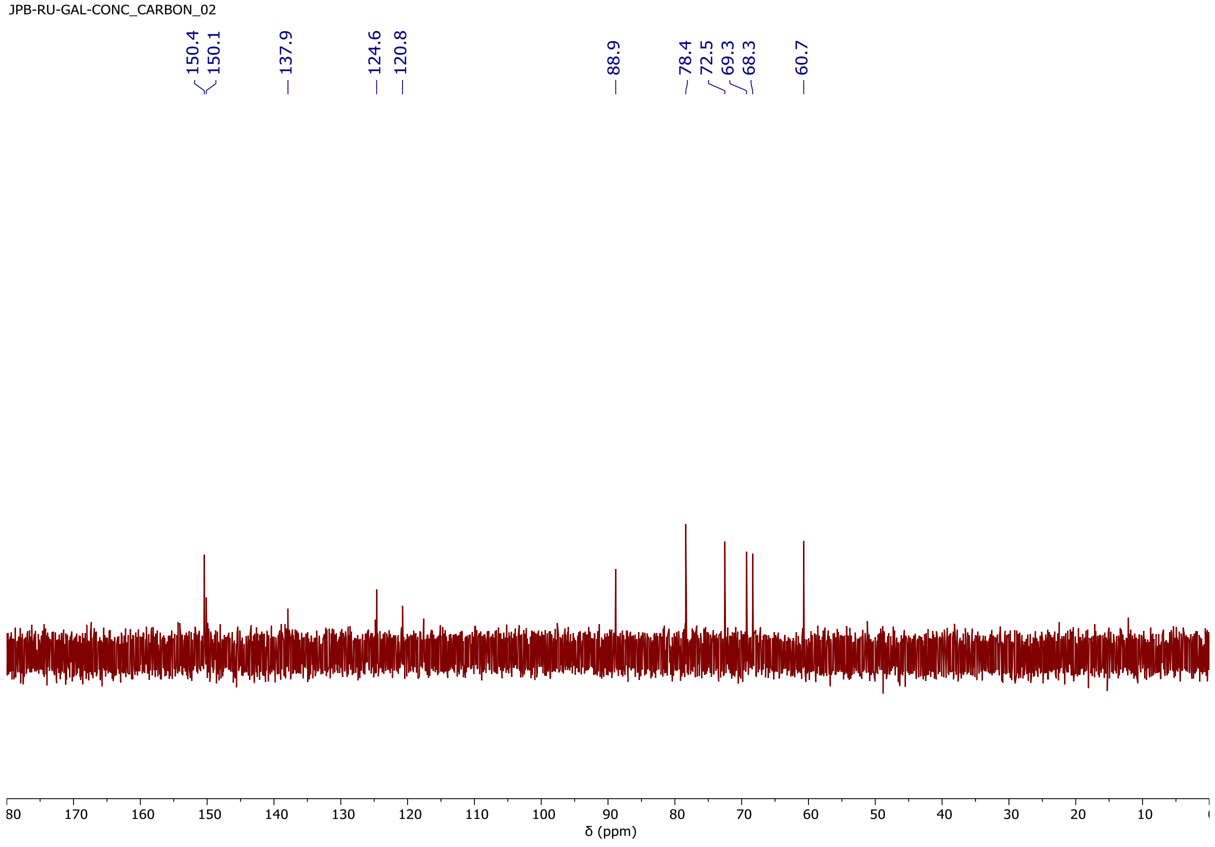

Figure S19. *^{13}C NMR spectrum (D_2O, 126 MHz) of **7Gal***

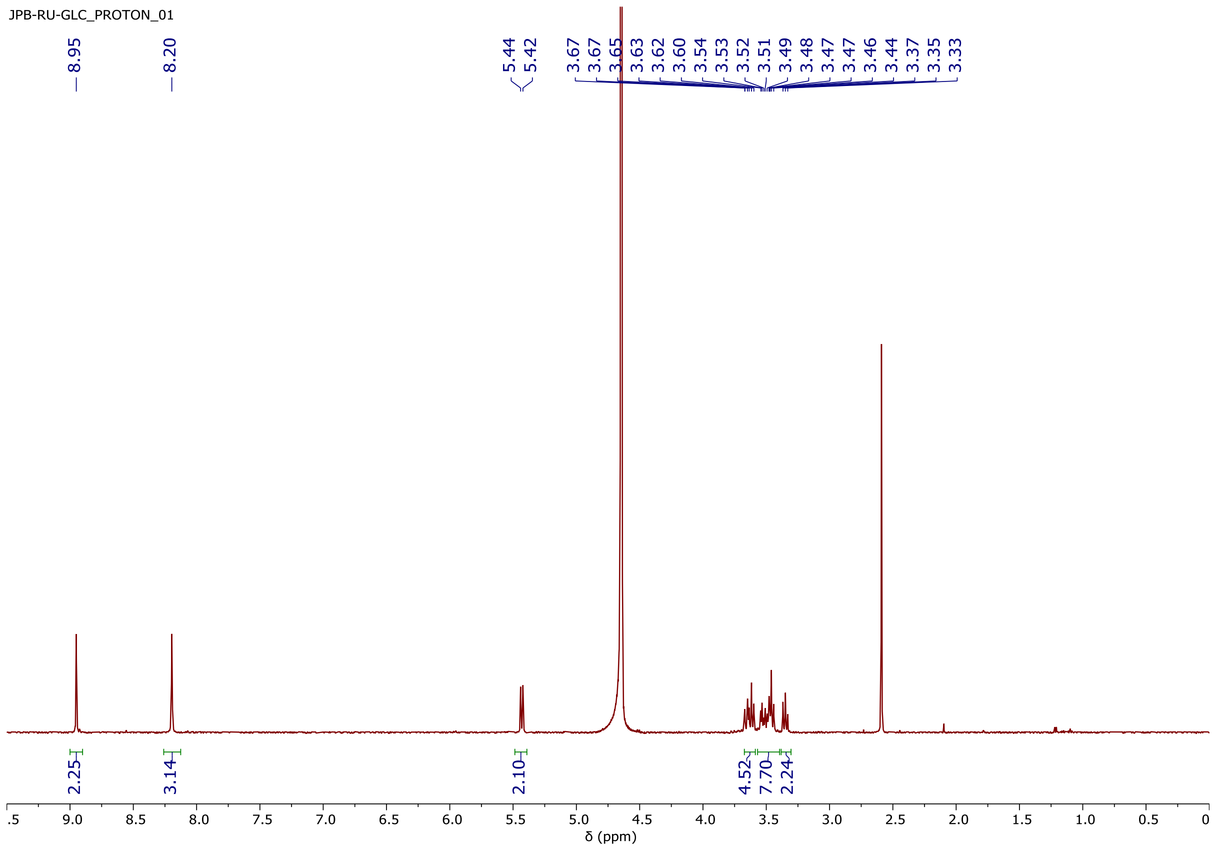

Figure S20. *^{1}H NMR spectrum (D_2O, 500 MHz) of* ***7Glc***

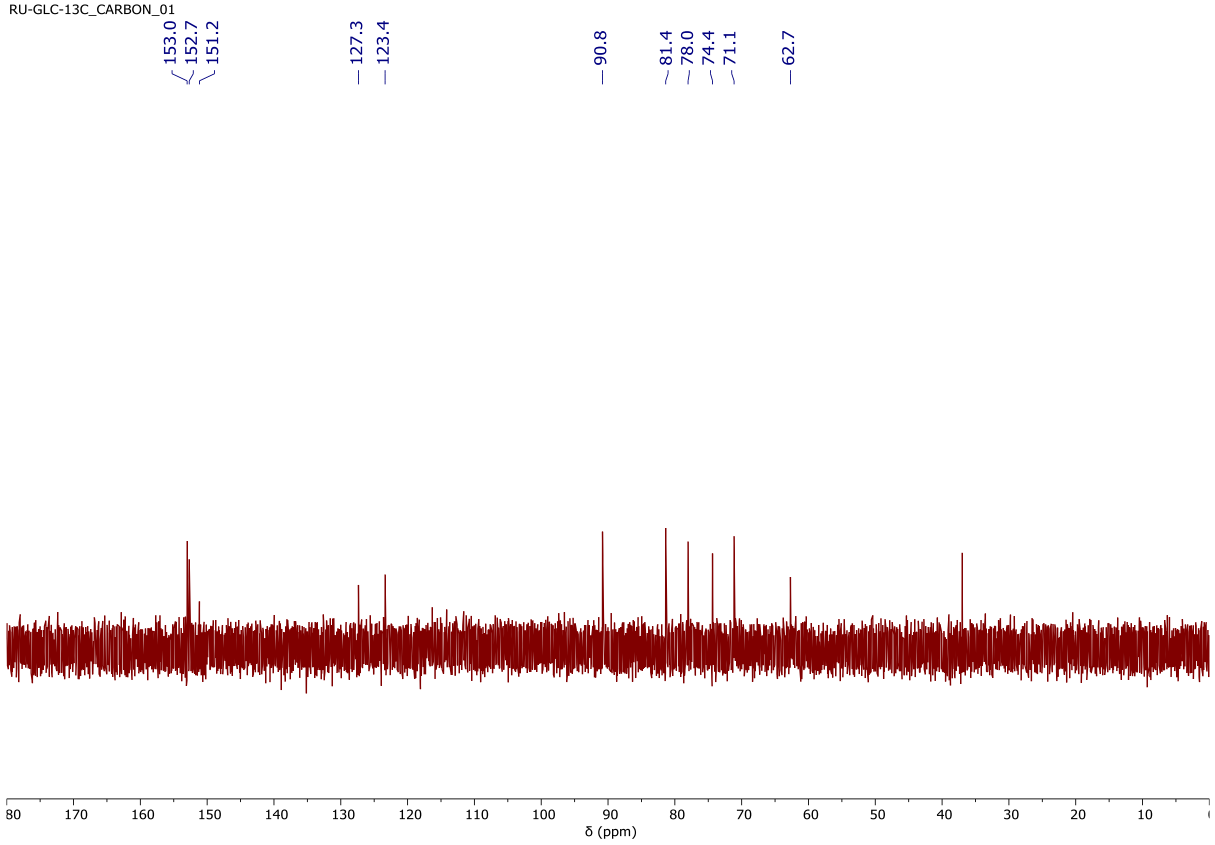

Figure S21. *^{13}C NMR spectrum (D_2O, 126 MHz) of* ***7Glc***

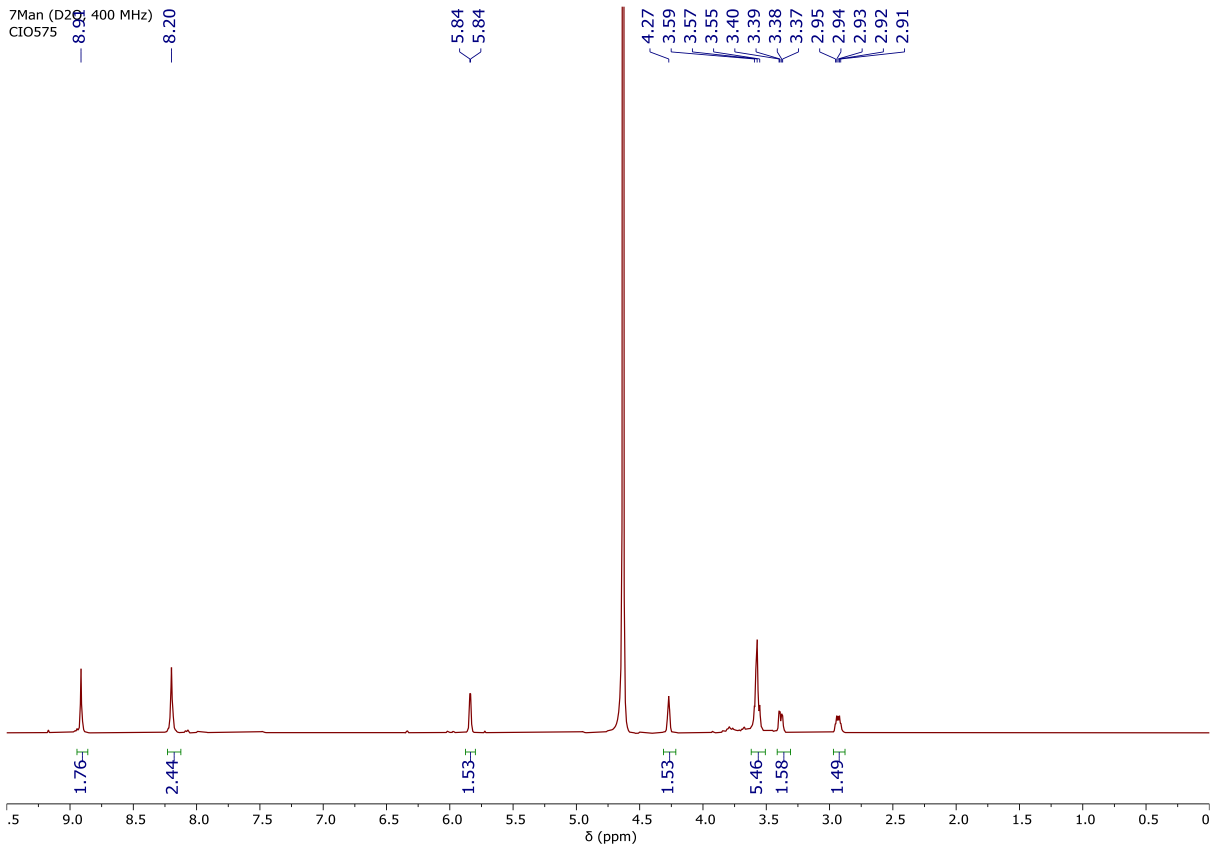

Figure S22. *^{1}H NMR spectrum (D_2O, 400 MHz) of* ***7Man***

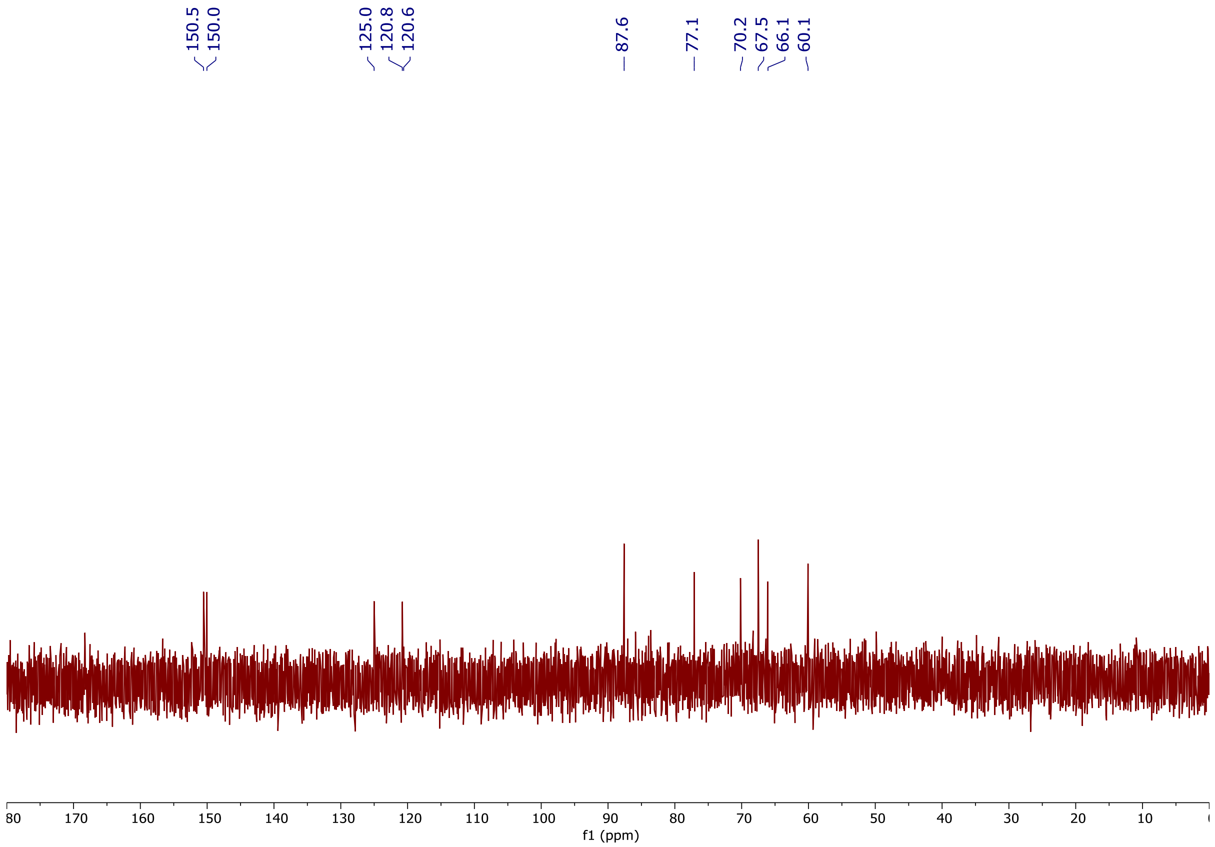

Figure S23. *^{13}C NMR spectrum (D_2O, 101 MHz) of* ***7Man***

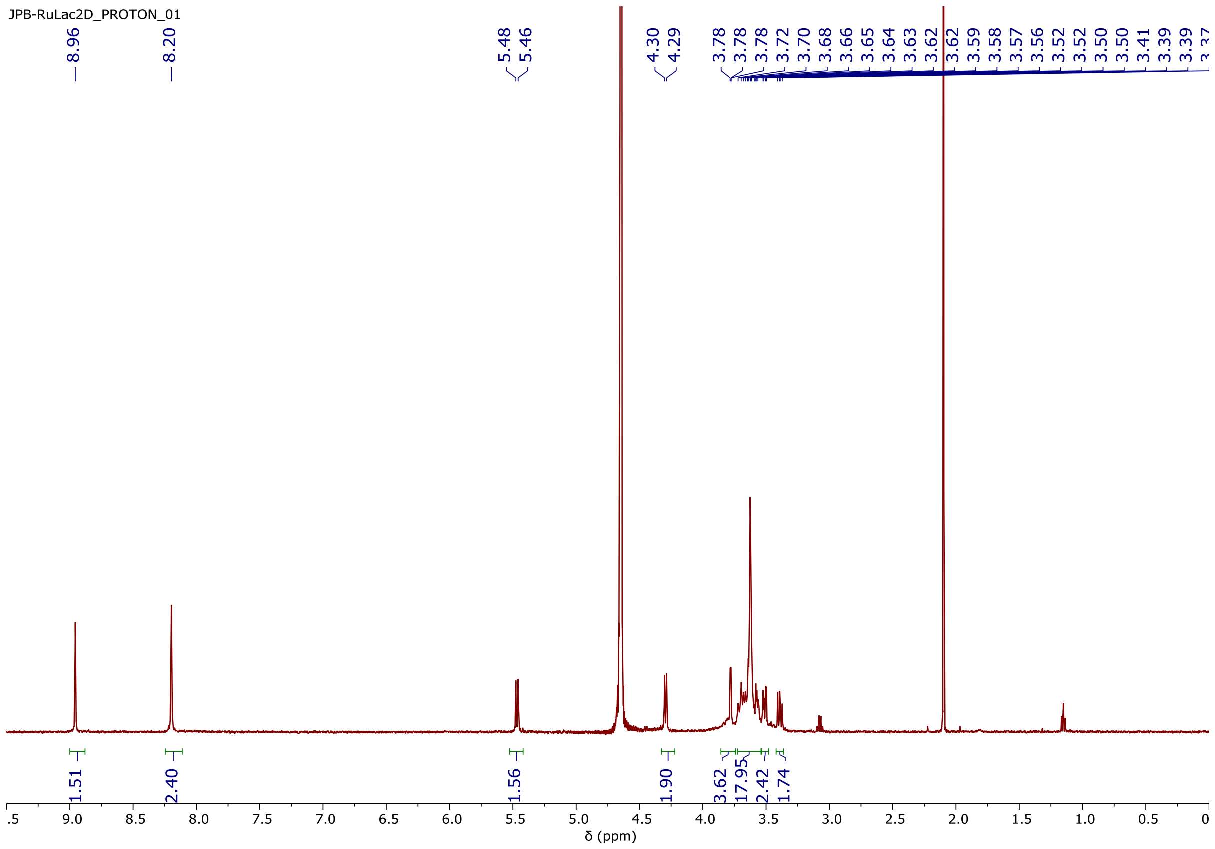

Figure S24. *^{1}H NMR spectrum (D_2O, 500 MHz) of* ***7Lac***

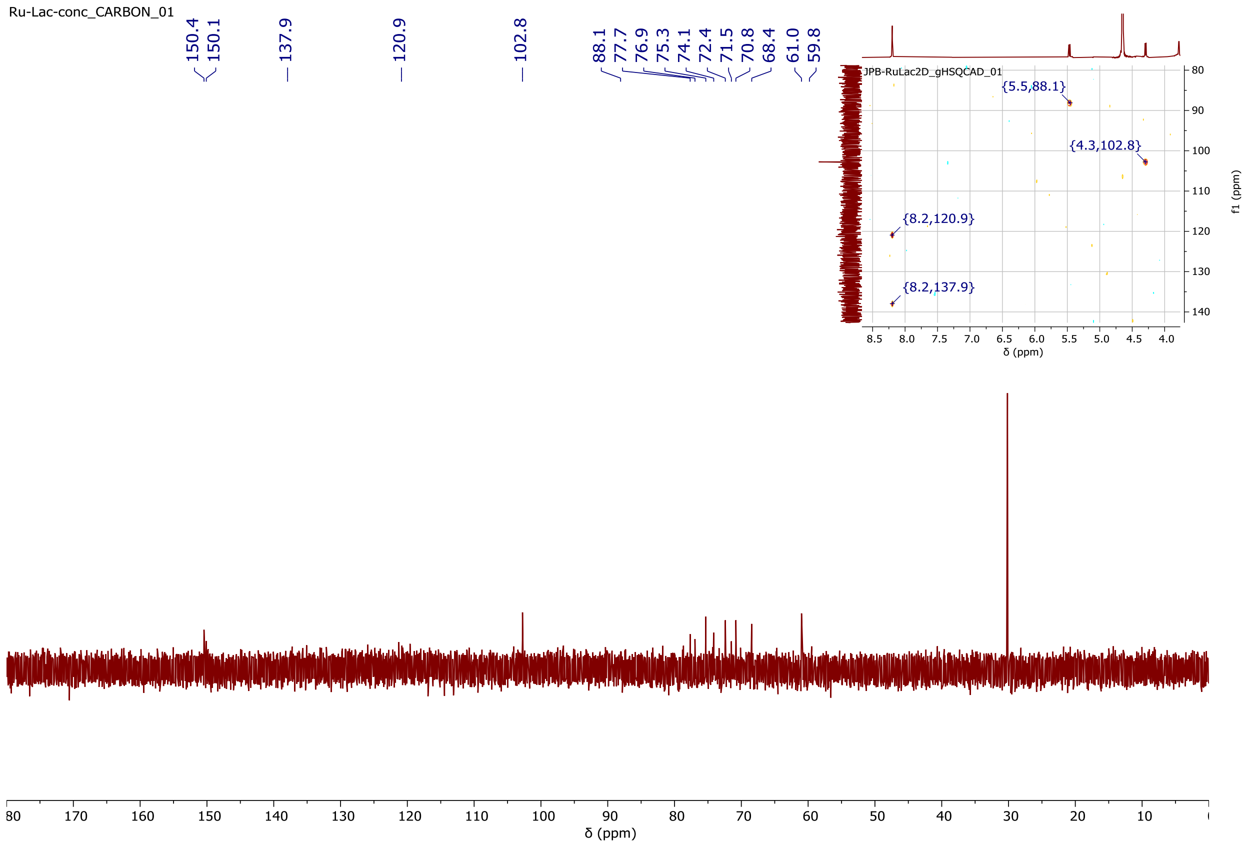

Figure S25. *^{13}C NMR spectrum (D_2O, 126 MHz) of* ***7Lac*** *(inset: detail of HSQC)*

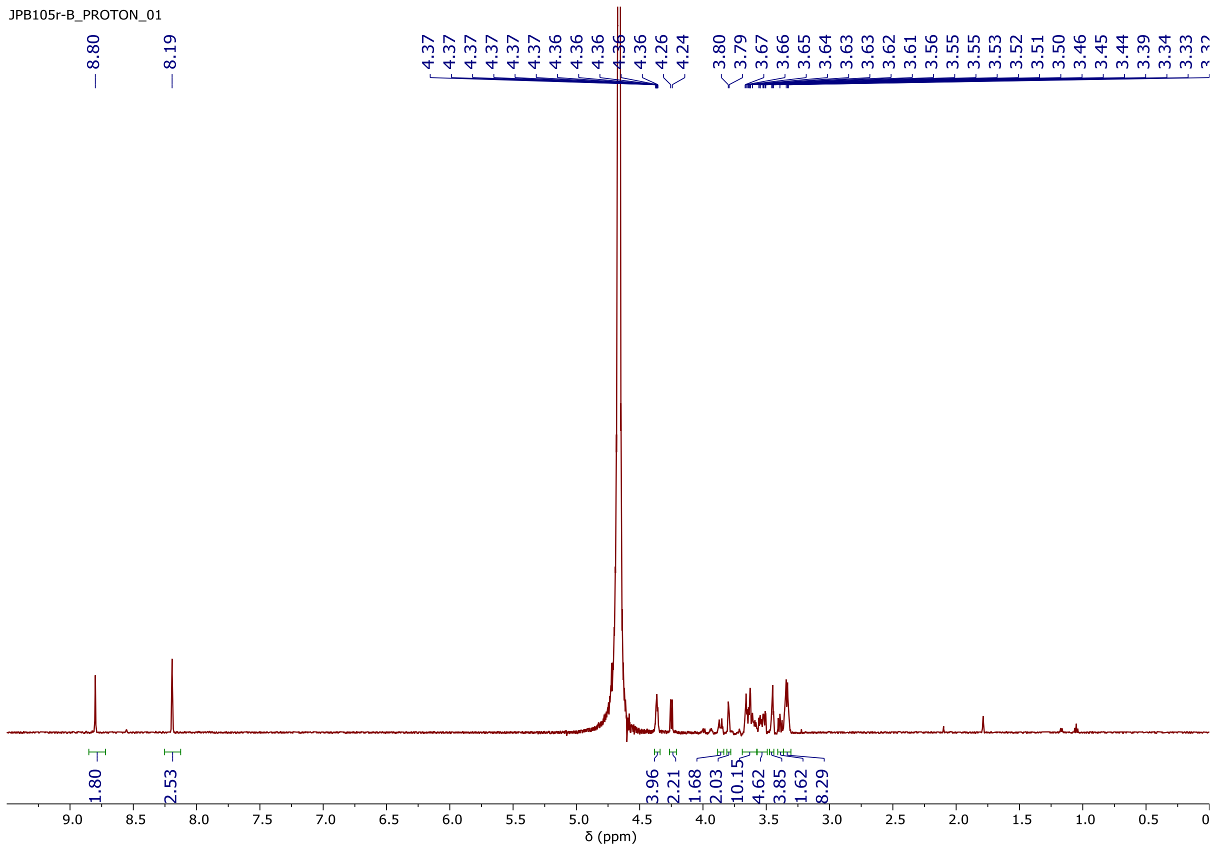

Figure S26. ^{1}H NMR spectrum (D_2O, 600 MHz) of ***8Gal***

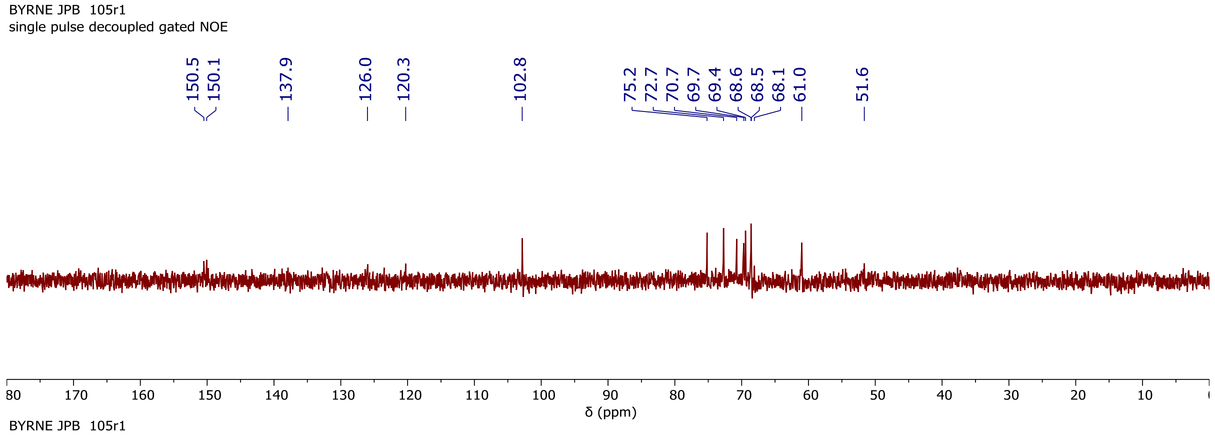

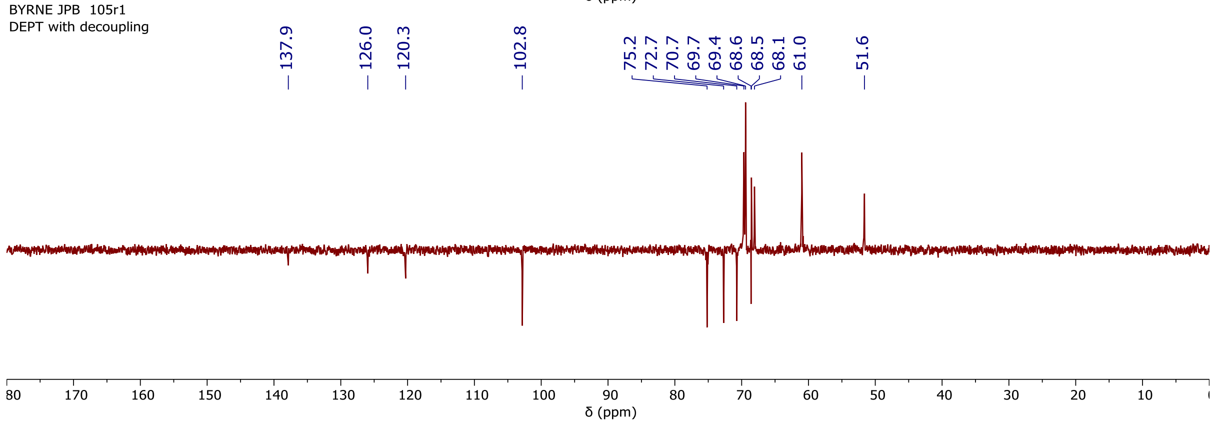

Figure S27. ^{13}C NMR spectrum and DEPT (D_2O, 101 MHz) of ***8Gal***

Biofilm Formation Assay for Ligand 6Gal

As a control experiment, the ability of ligand **6Gal**, (the precursor to complex **8Gal**) which showed anti-biofilm activity was also assessed. The ligand did not show any statistically significant inhibition of biofilm formation by PAO1 under conditions analogous to those described in the main body of the manuscript[S1].

[S1] G. A. O'Toole, *J. Vis. Exp.* 2011, *47*, 2437.

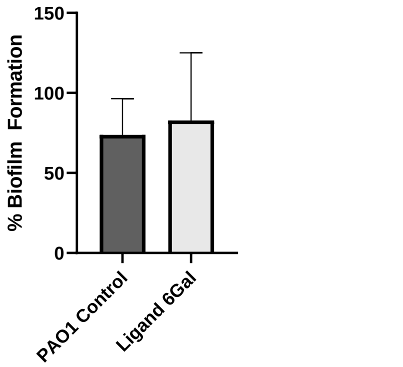

Figure S28. *Percentage biofilm formation determined by crystal violet staining of biofilms (absorbance measured at 590 nm).*

Minimum Inhibitory Concentration (MIC) Testing

Ligand **6Gal** and complexes **7** and **8Gal** were tested for their ability to inhibit growth of *P. aeruginosa* (PAO1) at various concentrations. In brief: PAO1 was seeded into wells at 10^6 CFU/ml. Serial dilutions of each of the compounds was then added to the wells and a set of control wells with no compound was also set up. The plates were incubated for 24hrs at 37°C before absorption readings at 600nm were taken. The Minimum Inhibitory Concentration (MIC) is defined as the lowest concentration of an antimicrobial agent that inhibits the visible *in-vitro* growth of microorganisms. Each experiment was performed in triplicate. Compounds at high concentration were very coloured but turbidity could be observed in all wells (See images alongside MIC graphs below).

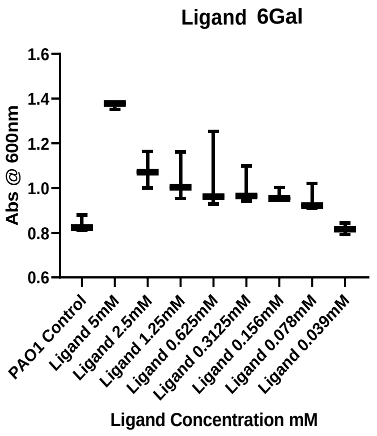

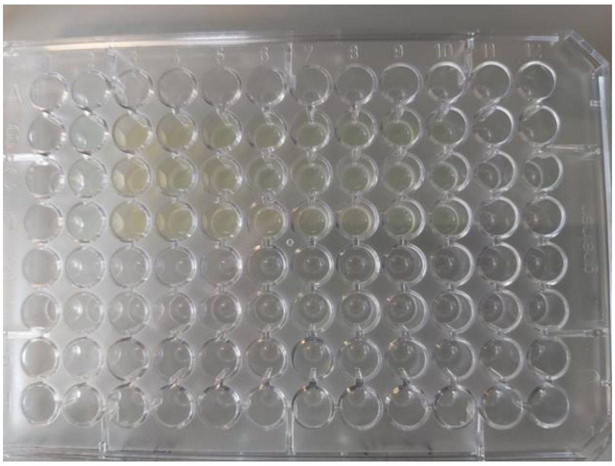

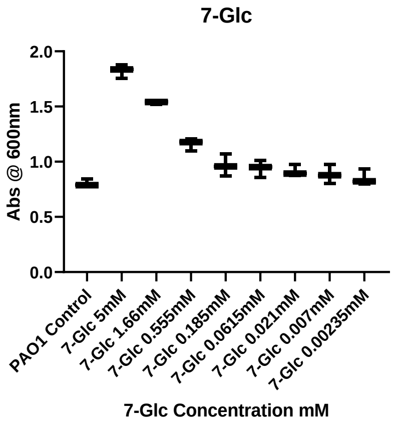

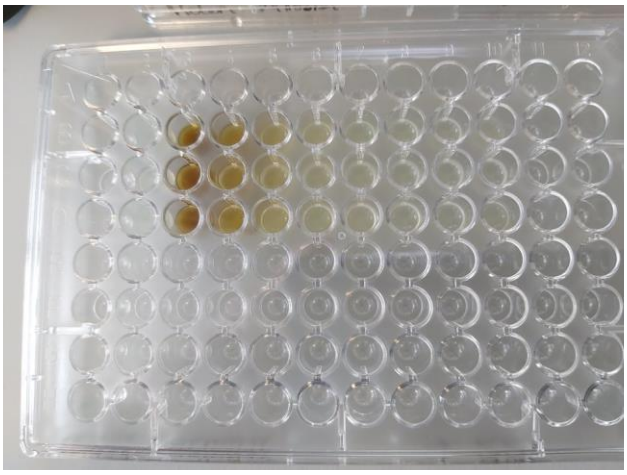

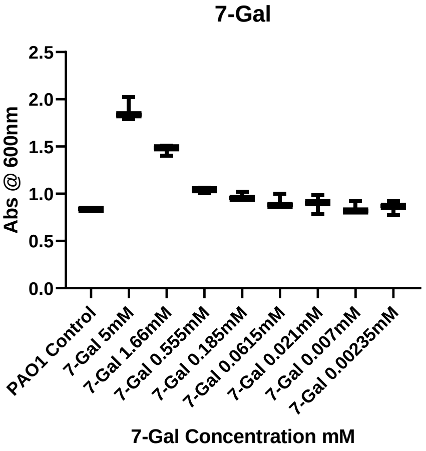
7-Gal
Abs @ 600nm
0.0
0.5
1.0
1.5
2.0
2.5
PAO1 Control
7-Gal 5mM
7-Gal 1.66mM
7-Gal 0.555mM
7-Gal 0.185mM
7-Gal 0.0615mM
7-Gal 0.021mM
7-Gal 0.007mM
7-Gal 0.00235mM
7-Gal Concentration mM

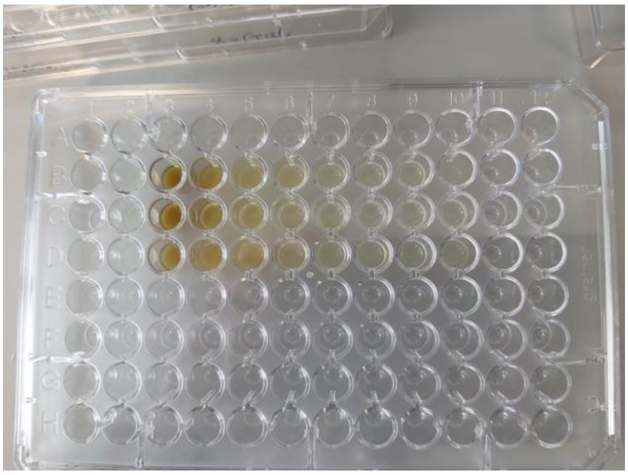

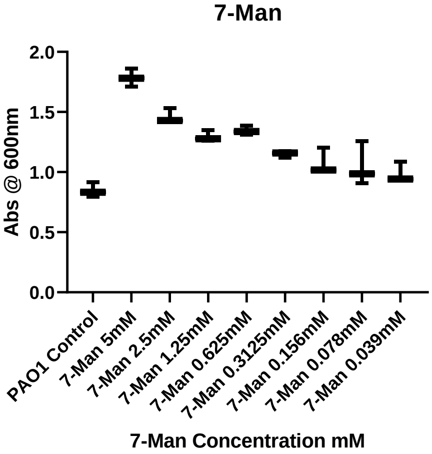
7-Man
Abs @ 600nm
0.0
0.5
1.0
1.5
2.0
PAO1 Control
7-Man 5mM
7-Man 2.5mM
7-Man 1.25mM
7-Man 0.625mM
7-Man 0.3125mM
7-Man 0.156mM
7-Man 0.078mM
7-Man 0.039mM
7-Man Concentration mM

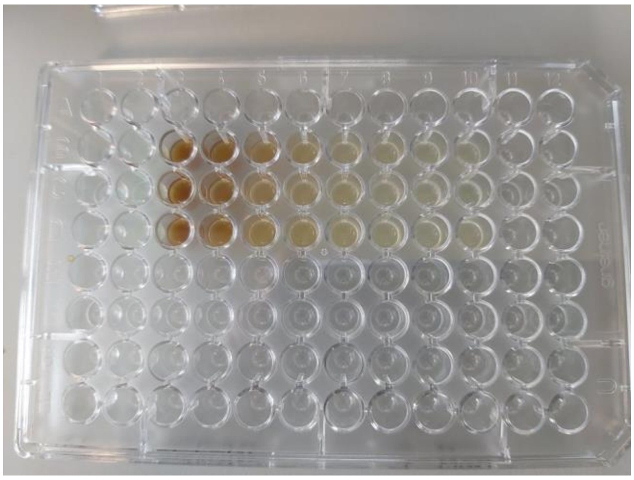

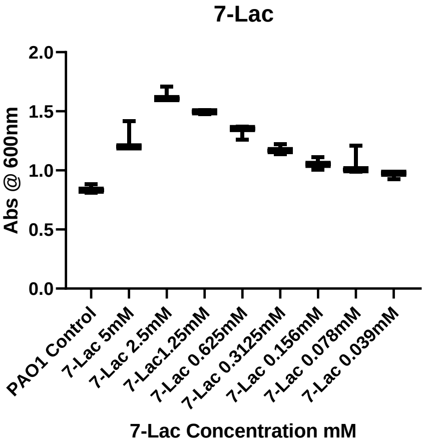
7-Lac
Abs @ 600nm
0.0
0.5
1.0
1.5
2.0
PAO1 Control
7-Lac 5mM
7-Lac 2.5mM
7-Lac1.25mM
7-Lac 0.625mM
7-Lac 0.3125mM
7-Lac 0.156mM
7-Lac 0.078mM
7-Lac 0.039mM
7-Lac Concentration mM

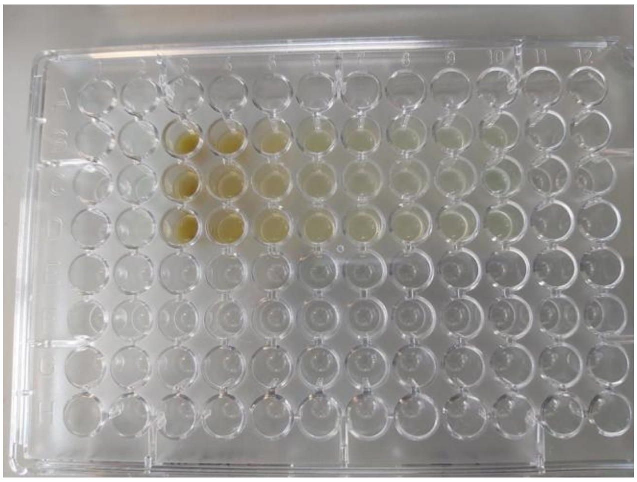

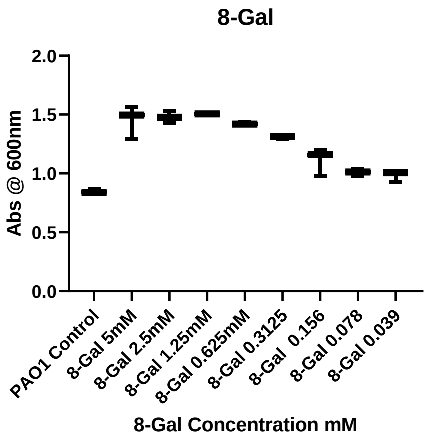

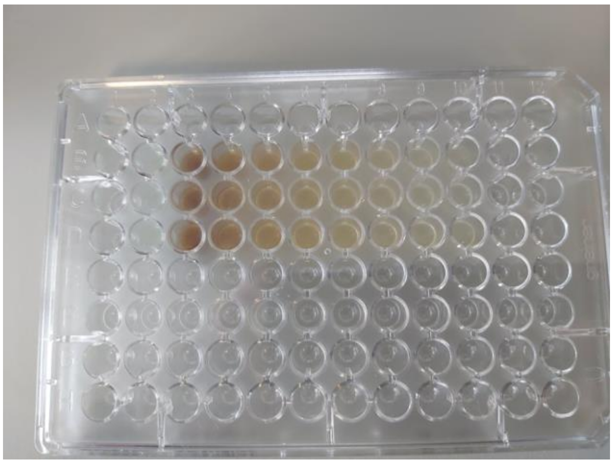

Minimum Bactericidal Concentration (MBC) Testing

After 24 hours incubation of PAO1 with various concentrations of ligand **6Gal** or complexes **7** and **8Gal**, samples from wells were applied to TSA (Tryptic Soy Agar) for culturing colonies. Since turbidity was clear in all wells, only the three highest compound concentration wells for each compound were plated out. The Minimum bactericidal concentration (MBC) is defined as the lowest concentration of antibiotic that kills 99.9% of the inoculum. It is determined by subculturing the last clear MIC tube onto growth medium and examining for bacterial growth. All plates returned TNTC (Too numerous to count) meaning none of the compounds tested had any bactericidal effect on PAO1 (*Pseudomonas aeruginosa*).

Table S1 *Summary of results from MBC Testing for compounds* ***6Gal***, ***7*** *and* ***8Gal***.

	**Concentration (mM)**				
	5	2.5	1.66	1.25	0.55
**7Glc**	TNTC	–	TNTC	–	TNTC
**7Gal**	TNTC	–	TNTC	–	TNTC
**7-Man**	TNTC	TNTC	–	TNTC	–
**7-Lac**	TNTC	TNTC	–	TNTC	–
**8-Gal**	TNTC	TNTC	–	TNTC	–
**6Gal** (ligand)	TNTC	TNTC	–	TNTC	–

HeLa Cytotoxicity Testing

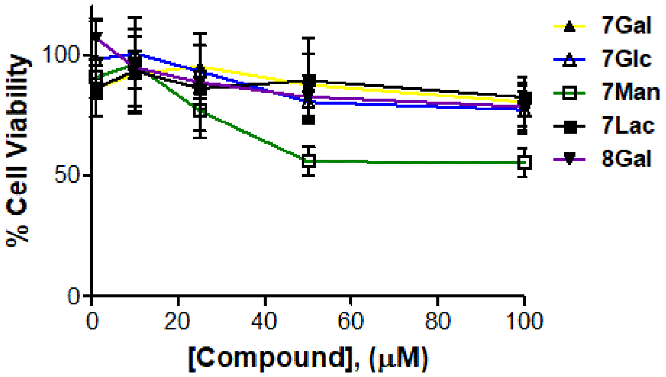

Figure S29. *HeLa cell viability upon incubation with complexes **7** and **8Gal**.*

Complexes **7** and **8Gal** are non-toxic to HeLa cells. HeLa cells were treated for 24h with a range of concentrations of the indicated compounds in a 96-well plate. After the required incubation period, alamar blue dye (20 µL) was added to each well and samples were incubated for 4h. Values represent the mean ± S.E.M. of two independent experiments performed in triplicate. Cytotoxicity testing was carried out by Dr Sandra Bright (Trinity Biomedical Sciences Institute, Trinity College Dublin, Ireland) as described below.

Cell culture: HeLa (human cervical cancer) cells were grown in Dulbecco's Modified Eagle Medium (Glutamax) supplemented with 10% fetal bovine serum and 50 µg/ml penicillin/streptomycin at 37˚C in a humidified atmosphere of 5% CO_2.

Alamar blue viability assay: HeLa cells were seeded at a density of $5x10^3$ cells/well in 96-well plates and treated with the indicated compounds for 24h. Alamar blue (20 µL) was then added to each well and incubated at 37°C in the dark for 4h. Plates were then read on a fluorescence plate reader (SpectraMax Gemini, Molecular Devices) with excitation and emission wavelengths of 544nm and 590nm respectively. Activity is expressed as percentage cell viability compared to vehicle treated controls. All data points (expressed as means ± S.E.M.) were analysed using GRAPHPAD Prism (Graphpad software Inc., San Diego, CA).

Supplementary X-ray crystallography data

Table S2 *Bond lengths [Å] and angles [°] of supramolecular and non-classical hydrogen bonding interactions in the structure of **3Gal** (atoms labelled in image below).*

D—H···*A*	*D*—H	H···*A*	*D*···*A*	∠*D*—H···*A*
C8—H8···O17	1.00	2.66	3.572(10)	151.7
C5—H5···N5	0.95	2.61	3.511(10)	157.7
C35—H35B···O7	0.98	2.78	3.545(11)	135.4
C19—H19A···O1	0.98	2.80	3.496(10)	128.4
C19—H19B···N2	0.98	2.59	3.537(11)	163.5
C24—H24···N1	0.95	2.58	3.482(9)	158.1
C38—H38C···O2	0.98	2.78	3.547(12)	135.2
C38—H38B···N6	0.98	2.64	3.363(11)	131.1
C16—H16B···O16	0.98	2.68	3.539(10)	146.7
C16—H16C···O18	0.98	2.74	3.411(12)	126.5
C29—H29···O5	1.00	2.49	3.417(10)	155.0
C31—H31C···O16	0.98	2.66	3.457(12)	138.8
C14—H14A···O4	0.98	2.66	3.540(10)	149.5
C21—H21···O18	0.95	2.48	3.334(12)	149.0

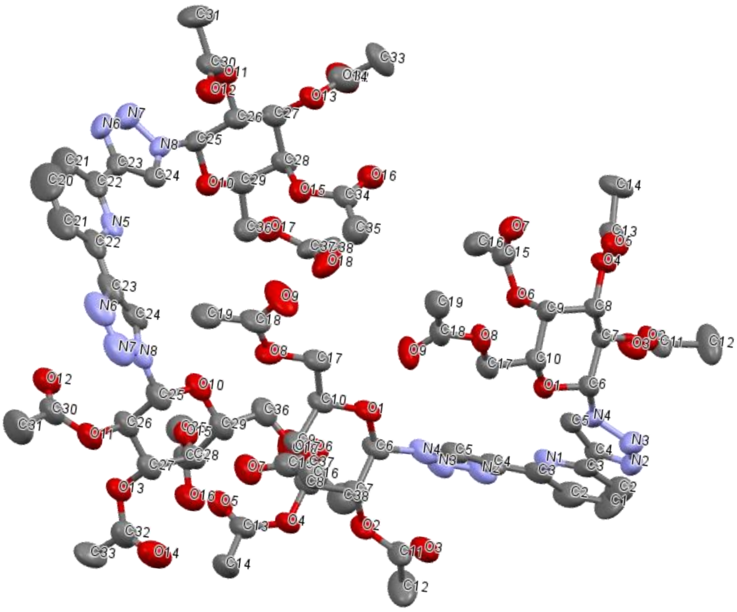

checkCIF/PLATON report

Structure factors have been supplied for datablock(s) sal27

THIS REPORT IS FOR GUIDANCE ONLY. IF USED AS PART OF A REVIEW PROCEDURE FOR PUBLICATION, IT SHOULD NOT REPLACE THE EXPERTISE OF AN EXPERIENCED CRYSTALLOGRAPHIC REFEREE.

No syntax errors found. CIF dictionary Interpreting this report

Datablock: sal27

```
Bond precision:  C-C = 0.0123 A          Wavelength=1.54178

Cell:             a=13.033(15)      b=13.033(15)      c=44.95(5)
                  alpha=90          beta=90           gamma=120
Temperature:      100 K

                Calculated                  Reported
Volume          6612(20)                    6613(17)
Space group     P 32 2 1                    P 32 2 1
Hall group      P 32 2"                     P 32 2"
Moiety formula  C37 H43 N7 O18 [+ solvent] 2(C18.5 H21.5 N3.5 O9)
Sum formula     C37 H43 N7 O18 [+ solvent] C37 H43 N7 O18
Mr              873.78                      873.78
Dx,g cm-3       1.317                       1.316
Z               6                           6
Mu (mm-1)       0.911                       0.911
F000            2748.0                      2748.0
F000’           2758.16
h,k,lmax        14,14,50                    14,14,49
Nref            6370[ 3661]                 6347
Tmin,Tmax       0.774,0.847
Tmin’           0.724

Correction method= Not given

Data completeness= 1.73/1.00      Theta(max)= 59.042

R(reflections)= 0.0618( 5522)     wR2(reflections)= 0.1507( 6347)

S = 1.075                  Npar= 569
```

The following ALERTS were generated. Each ALERT has the format
test-name_ALERT_alert-type_alert-level.
Click on the hyperlinks for more details of the test.

Alert level B

THETM01_ALERT_3_B The value of sine(theta_max)/wavelength is less than 0.575
Calculated sin(theta_max)/wavelength = 0.5562

Author Response: The reflectivity of this sample was weak, therefore copper wavelength was used in order to improve the data quality. The resolution was pushed to the limit but even though the resolution was limited.

PLAT340_ALERT_3_B Low Bond Precision on C-C Bonds .... 0.01229 Ang.

Author Response: This is linked to the limited resolution that could be achieved with the existing samples.

PLAT411_ALERT_2_B Short Inter H...H Contact H1 ..H20 . 1.84 Ang.
1+x,-1+y,z = 1_645 Check

Author Response: These atoms belong to the pyridyne hydrogen in position 4. These atoms clearly belong there despite their closeness.

PLAT411_ALERT_2_B Short Inter H...H Contact H1 ..H20 . 1.84 Ang.
2+x-y,1-y,4/3-z = 4_766 Check

Author Response: These atoms belong to the pyridyne hydrogen in position 4. These atoms clearly belong there despite their closeness.

Alert level C

PLAT089_ALERT_3_C Poor Data / Parameter Ratio (Zmax < 18) .... 6.42 Note
PLAT148_ALERT_3_C s.u. on the a - Axis is (Too) Large .... 0.015 Ang.
PLAT148_ALERT_3_C s.u. on the c - Axis is (Too) Large .... 0.050 Ang.
PLAT213_ALERT_2_C Atom C20 has ADP max/min Ratio ..... 3.2 prolat
PLAT234_ALERT_4_C Large Hirshfeld Difference C37 --C38 . 0.16 Ang.
PLAT242_ALERT_2_C Low 'MainMol' Ueq as Compared to Neighbors of C11 Check
PLAT242_ALERT_2_C Low 'MainMol' Ueq as Compared to Neighbors of C18 Check
PLAT250_ALERT_2_C Large U3/U1 Ratio for Average U(i,j) Tensor .... 2.3 Note
PLAT911_ALERT_3_C Missing FCF Refl Between Thmin & STh/L= 0.556 12 Report

---

Alert level G

PLAT042_ALERT_1_G Calc. and Reported MoietyFormula Strings Differ Please Check
PLAT083_ALERT_2_G SHELXL Second Parameter in WGHT Unusually Large 7.10 Why ?
PLAT152_ALERT_1_G The Supplied and Calc. Volume s.u. Differ by ... 3 Units
PLAT432_ALERT_2_G Short Inter X...Y Contact O14 ..C12 2.99 Ang.
y,x,1-z = 6_556 Check
PLAT606_ALERT_4_G VERY LARGE Solvent Accessible VOID(S) in Structure ! Info
PLAT791_ALERT_4_G Model has Chirality at C6 (Sohnke SpGr) R Verify
PLAT791_ALERT_4_G Model has Chirality at C7 (Sohnke SpGr) R Verify

```
PLAT791_ALERT_4_G Model has Chirality at C8         (Sohnke SpGr)          S Verify
PLAT791_ALERT_4_G Model has Chirality at C9         (Sohnke SpGr)          S Verify
PLAT791_ALERT_4_G Model has Chirality at C10        (Sohnke SpGr)          R Verify
PLAT791_ALERT_4_G Model has Chirality at C25        (Sohnke SpGr)          R Verify
PLAT791_ALERT_4_G Model has Chirality at C26        (Sohnke SpGr)          R Verify
PLAT791_ALERT_4_G Model has Chirality at C27        (Sohnke SpGr)          S Verify
PLAT791_ALERT_4_G Model has Chirality at C28        (Sohnke SpGr)          S Verify
PLAT791_ALERT_4_G Model has Chirality at C29        (Sohnke SpGr)          R Verify
PLAT868_ALERT_4_G ALERTS Due to the Use of _smtbx_masks Suppressed         ! Info
PLAT909_ALERT_3_G Percentage of I>2sig(I) Data at Theta(Max) Still       53% Note
PLAT933_ALERT_2_G Number of OMIT Records in Embedded .res File ...         1 Note
PLAT978_ALERT_2_G Number C-C Bonds with Positive Residual Density.         0 Info
```

```
  0 ALERT level A = Most likely a serious problem - resolve or explain
  4 ALERT level B = A potentially serious problem, consider carefully
  9 ALERT level C = Check. Ensure it is not caused by an omission or oversight
 19 ALERT level G = General information/check it is not something unexpected

  2 ALERT type 1 CIF construction/syntax error, inconsistent or missing data
 10 ALERT type 2 Indicator that the structure model may be wrong or deficient
  7 ALERT type 3 Indicator that the structure quality may be low
 13 ALERT type 4 Improvement, methodology, query or suggestion
  0 ALERT type 5 Informative message, check
```

It is advisable to attempt to resolve as many as possible of the alerts in all categories. Often the minor alerts point to easily fixed oversights, errors and omissions in your CIF or refinement strategy, so attention to these fine details can be worthwhile. In order to resolve some of the more serious problems it may be necessary to carry out additional measurements or structure refinements. However, the purpose of your study may justify the reported deviations and the more serious of these should normally be commented upon in the discussion or experimental section of a paper or in the "special_details" fields of the CIF. checkCIF was carefully designed to identify outliers and unusual parameters, but every test has its limitations and alerts that are not important in a particular case may appear. Conversely, the absence of alerts does not guarantee there are no aspects of the results needing attention. It is up to the individual to critically assess their own results and, if necessary, seek expert advice.

Publication of your CIF in IUCr journals

A basic structural check has been run on your CIF. These basic checks will be run on all CIFs submitted for publication in IUCr journals (*Acta Crystallographica*, *Journal of Applied Crystallography*, *Journal of Synchrotron Radiation*); however, if you intend to submit to *Acta Crystallographica Section C* or *E* or *IUCrData*, you should make sure that full publication checks are run on the final version of your CIF prior to submission.

Publication of your CIF in other journals

Please refer to the *Notes for Authors* of the relevant journal for any special instructions relating to CIF submission.

PLATON version of 22/04/2020; check.def file version of 09/03/2020

Datablock sal27 - ellipsoid plot

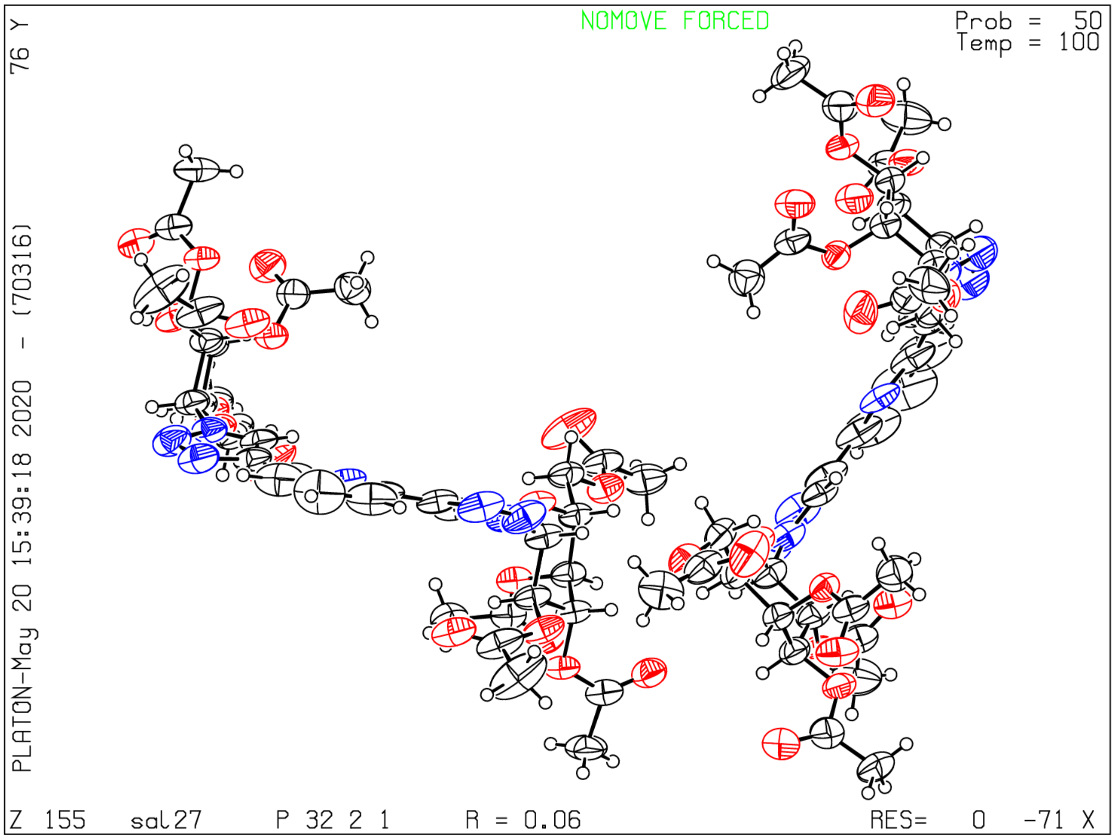

Supplement: RA-011-D0RA05107A-s001 [file RA-011-D0RA05107A-s001.pdf]
